# Mandatory, voluntary, repetitive, or one-off post-migration follow-up for tuberculosis prevention and control: A systematic review

**DOI:** 10.1371/journal.pmed.1004030

**Published:** 2023-01-31

**Authors:** Katharina Wahedi, Dominik Zenner, Sergio Flores, Kayvan Bozorgmehr

**Affiliations:** 1 Section for Health Equity Studies & Migration, Department of General Practice & Health Services Research, Heidelberg University Hospital, Marsilius-Arkaden, Heidelberg, Germany; 2 Clinical Reader in Infectious Disease Epidemiology, Wolfson Institute of Population Health, Queen Mary University of London, London, United Kingdom; 3 Department of Public Healthy and Caring Sciences, Child Health and Parenting (CHAP), Uppsala University, Uppsala, Sweden; 4 Department of Population Medicine and Health Services Research, School of Public Health, Bielefeld University, Germany, Bielefeld, Germany; PLOS Medicine Editorial Board, UNITED STATES

## Abstract

**Background:**

Post-migration follow-up of migrants identified to be at-risk of developing tuberculosis during the initial screening is effective, but programmes vary across countries. We aimed to review main strategies applied to design follow-up programmes and analyse the effect of key programme characteristics on reported coverage (i.e., proportion of migrants screened among those eligible for screening) or yields (i.e., proportion of active tuberculosis among those identified as eligible for follow-up screening).

**Methods and findings:**

We performed a systematic review and meta-analysis of studies reporting yields of follow-up screening programmes. Studies were included if they reported the rate of tuberculosis disease detected in international migrants through active case finding strategies and applied a post-migration follow-up (defined as one or more additional rounds of screening after finalising the initial round). For this, we retrieved all studies identified by Chan and colleagues for their systematic review (in their search until January 12, 2017) and included those reporting from active follow-up programmes. We then updated the search (from January 12, 2017 to September 30, 2022) using Medline and Embase via Ovid. Data were extracted on reported coverage, yields, and key programme characteristics, including eligible population, mode of screening, time intervals for screening, programme providers, and legal frameworks. Differences in follow-up programmes were tabulated and synthesised narratively. Meta-analyses in random effect models and exploratory analysis of subgroups showed high heterogeneity (I^2^ statistic > 95.0%). We hence refrained from pooling, and estimated yields and coverage with corresponding 95% confidence intervals (CIs), stratified by country, legal character (mandatory versus voluntary screening), and follow-up scheme (one-off versus repetitive screening) using forest plots for comparison and synthesis. Of 1,170 articles, 24 reports on screening programmes from 7 countries were included, with considerable variation in eligible populations, time intervals of screening, and diagnostic protocols. Coverage varied, but was higher than 60% in 15 studies, and tended to be lower in voluntary compared to compulsory programmes, and higher in studies from the United States of America, Israel, and Australia. Yield varied within and between countries and ranged between 53.05 (31.94 to 82.84) in a Dutch study and 5,927.05 (4,248.29 to 8,013.71) in a study from the United States. Of 15 estimates with narrow 95% CIs for yields, 12 were below 1,500 cases per 100,000 eligible migrants. Estimates of yields in one-off follow-up programmes tended to be higher and were surrounded by less uncertainty, compared to those in repetitive follow-up programmes. Yields in voluntary and mandatory programmes were comparable in magnitude and uncertainty. The study is limited by the heterogeneity in the design of the identified screening programmes as effectiveness, coverage and yields also depend on factors often underreported or not known, such as baseline incidence in the respective population, reactivation rate, educative and administrative processes, and consequences of not complying with obligatory measures.

**Conclusion:**

Programme characteristics of post-migration follow-up screening for prevention and control of tuberculosis as well as coverage and yield vary considerably. Voluntary programmes appear to have similar yields compared with mandatory programmes and repetitive screening apparently did not lead to higher yields compared with one-off screening. Screening strategies should consider marginal costs for each additional round of screening.

## Abstract 2

In a systematic review, Dr Kayvan Bozorgmehr and colleagues explore mandatory, voluntary, repetitive or one-off post-migration follow-up for tuberculosis prevention and control.

## Author summary

**Why was this study done?:**

- Follow-up screening of migrants after an initial pre- or post- entry screening for tuberculosis has been shown to be effective to identify migrants suffering from tuberculosis that had either been missed in the initial screening or reactivated afterwards.
- However, little is known about the design and characteristics of the existing follow-up screening programmes and how these might affect outcome parameters, such as coverage (the proportion of migrants screened among those eligible for screening) and yields (proportion of active tuberculosis among those identified as eligible for follow-up screening).

**What did the researchers do and find?:**

- We performed a systematic review and identified 23 studies reporting data from follow-up screening programmes for tuberculosis in migrants.
- Despite high heterogeneity with respect to important programme characteristics, coverage was higher than 60% in the majority of studies and tended to be lower in voluntary compared to compulsory programmes.
- Yields were below 1,500 cases per 100,000 eligible migrants in the majority of studies with large sample sizes and tended to be higher in one-off compared to repetitive follow-up programmes, while being comparable between voluntary and mandatory programmes.

**What do these findings mean?:**

- Consensus is needed on what the best mode, timing, and criteria for follow-up screening among migrants are.
- Yield appears to be meaningfully high to justify follow-up programmes from a public health perspective.
- Mandatory or repetitive follow-up programmes do not appear to be superior to voluntary or one-off follow-up programmes with respect to yields.

## Introduction

Several countries with a low incidence of tuberculosis implement active case finding strategies for tuberculosis among immigrants for prevention and control of the disease. Active case finding is often used as a synonym to screening [1], but may also imply other proactive measures to identify tuberculosis disease among asymptomatic populations based on some measure of “risk.” This is different to passive strategies that test for tuberculosis only among symptomatic individuals seeking health care [1]. Active case finding strategies in the context of migration may comprise pre-entry, upon-entry, or post-entry screening programmes or a combination thereof [2]. As there is no strict definition of upon-entry versus post-entry screening, we refer to any screening measure performed after arrival as post-entry. As the incidence of tuberculosis among migrants is highest in the first 2 to 4 years after immigration [3], some active case finding strategies include a post-migration follow-up (hereafter referred to as “follow-up programme”).

A systematic review and meta-analysis by Chan and colleagues showed that post-migration follow-up of migrants identified as being at risk of developing TB disease during the initial screening resulted in high TB yields [4]. They reported a pooled cumulative incidence of 2,794 per 100,000 at-risk migrants (defined as migrants not diagnosed with TB disease, but showing either chest radiograph abnormalities, a history of TB disease, (latent) tuberculosis infection (TBI) or positive TB contact status) and a pooled incidence of 1,249 per 100,000 person-years of follow-up. Chan and colleagues argue that these incidences are not only substantially higher than the incidence of tuberculosis among migrants from high-incidence countries [5], but also higher than those from other high-risk groups, such as close or household contacts of tuberculosis patients [6] or patients with HIV infection [7].

However, screening strategies differ with respect to aims, scope, mode of implementation, legal character and diagnostic tests applied [8], and no studies have analysed differences in yields of screening by such key programme characteristics.

In order to inform post-migration follow-up programmes for tuberculosis prevention and control, we (i) systematically reviewed the literature of active case finding strategies with a post-migration follow-up component to provide an overview of their programme characteristics; and (ii) compared reported coverage and yields as well as key aspects such as voluntary versus mandatory screening and one-off versus repetitive rounds of follow-up screening.

## Methods

### Search strategy

Our search strategy comprised of 2 steps: First, we retrieved all studies identified by Chan and colleagues for their systematic review [4] and included those that reported the results of follow-up strategies, while excluding data from studies reporting passive case finding strategies only (see below). In a second step, we updated the search for data on post-migration follow-up programmes using the search strategy designed by Chan and colleagues in order to ensure that countries with more recently developed policies or recently published data from existing programmes would be included (for search protocol see Table A in S2 File). The systematic search included studies from January 12, 2017 (the day Chan and colleagues concluded their search) to September30, 2022 and was conducted using MEDLINE and Embase classic + Embase (via Ovid). Articles with abstracts available in English, French, German, or Spanish were considered. The review was not registered. This study is reported as per the Preferred Reporting Items for Systematic Reviews and Meta-Analyses (PRISMA) guideline (S1 File). All references were exported to EndNote version 7 and transferred to Excel. Titles and abstracts were screened in duplicate for a priori defined inclusion and exclusion criteria in line with the search strategy.

### Selection criteria

We included studies reporting on tuberculosis active case finding strategies, regardless of timing of the initial screening (pre-/post-entry) if they had a post-migration follow-up, and reported the rate of tuberculosis detected (TB disease per persons screened, or per persons identified for screening or per number of screenings, if provided) in international migrants, defined as “any person who is moving or has moved across an international border or within a state away from his/her habitual place of residence” [9]. We included primary studies and hand-searched reference lists of seminal papers and reviews and included eligible primary studies. We also searched key sources of grey literature, including conference abstracts and reports from key respiratory and infectious disease societies. We excluded studies with non-eligible study populations, those that reported on active case finding strategies but did not contain any follow-up, did not report any tuberculosis rates identified by their follow-up strategy, or reported on findings from passive case finding only. Any discrepancies between reviewers regarding study inclusion were resolved through discussion and by consensus among the reviewers and arbitration by KB and DZ when needed.

### Data analysis

Data was extracted on the timing of initial screening (pre- or post-entry), the eligible populations of migrants subject to follow-up screening, the diagnostics and means of follow-up (e.g., chest X-ray, clinical examination), the legal character of the programmes (voluntary or mandatory), the reported case finding rates of the follow-up, the follow-up rates among the eligible population (if reported), the actors and institutions involved in or in charge of executing the follow-up, and the time frame of follow-up. We differentiated between one-off follow-up, continuous follow-up after pre-entry screening (when a follow-up period, e.g., 2 years, is defined without further specifications), and repetitive rounds of follow-up (defined as repeated screenings performed in specified time intervals). Data extraction was conducted by KW and SF, checked for completeness and accuracy by KB, and revised or complemented, where needed by all authors.

Additionally, when the identified articles did not provide enough information for complete data extraction on the underlying screening policy, we complemented and triangulated the information by other sources and conducted specific searches on national screening policies, contacted authors of primary studies, searched the references of included articles and used original studies on policies, such as the survey of European Tuberculosis screening policies by Kunst and colleagues [8] and the ECDC public health guideline on screening newly arrived migrants [10].

### Coverage and yields

We calculated coverage as the proportion of migrants who presented for screening among all migrants identified as eligible for follow-up screening. As the definition of yield reported by the identified studies varied, we recalculated yields based on 3 different denominators to quantify the cascade of screening. Yields were defined as the fraction of active TB cases with TB disease among (i) those presented for follow-up screening (“screened-population-yield”); (ii) those identified as eligible for follow-up screening (“eligible-population-yield”); and (iii) the total population initially screened for eligibility (“whole-population-yield”).

### Synthesis

We tabulated key characteristics and provided a narrative synthesis of follow-up programmes.

A meta-analysis of coverage and yields with different denominators (screened-/eligible-/whole-population-yields) and exploratory stratification by country, programme characteristics (voluntary versus compulsory), and scheme of follow-up was performed. We used the I^2^ statistic to quantify heterogeneity, defined as the percentage of the variability in effect estimates that is due to heterogeneity rather than sampling error [11] and tested for heterogeneity between subgroups. Since heterogeneity was very high (I^2^ > 95% in all models and subgroups), limiting the validity of the meta-analysis, we refrained from pooling for the final synthesis presented in the results section. Results of the exploratory analysis can be found in Figs A–T in S2 File.

We report estimates of coverage and yields with corresponding exact binomial 95% confidence intervals (CIs) (following Clopper–Pearson procedures). Estimates were plotted and compared in forest plots, weighted by the inverse variance (which depends on the sample size).

## Results

**Fig 1:**
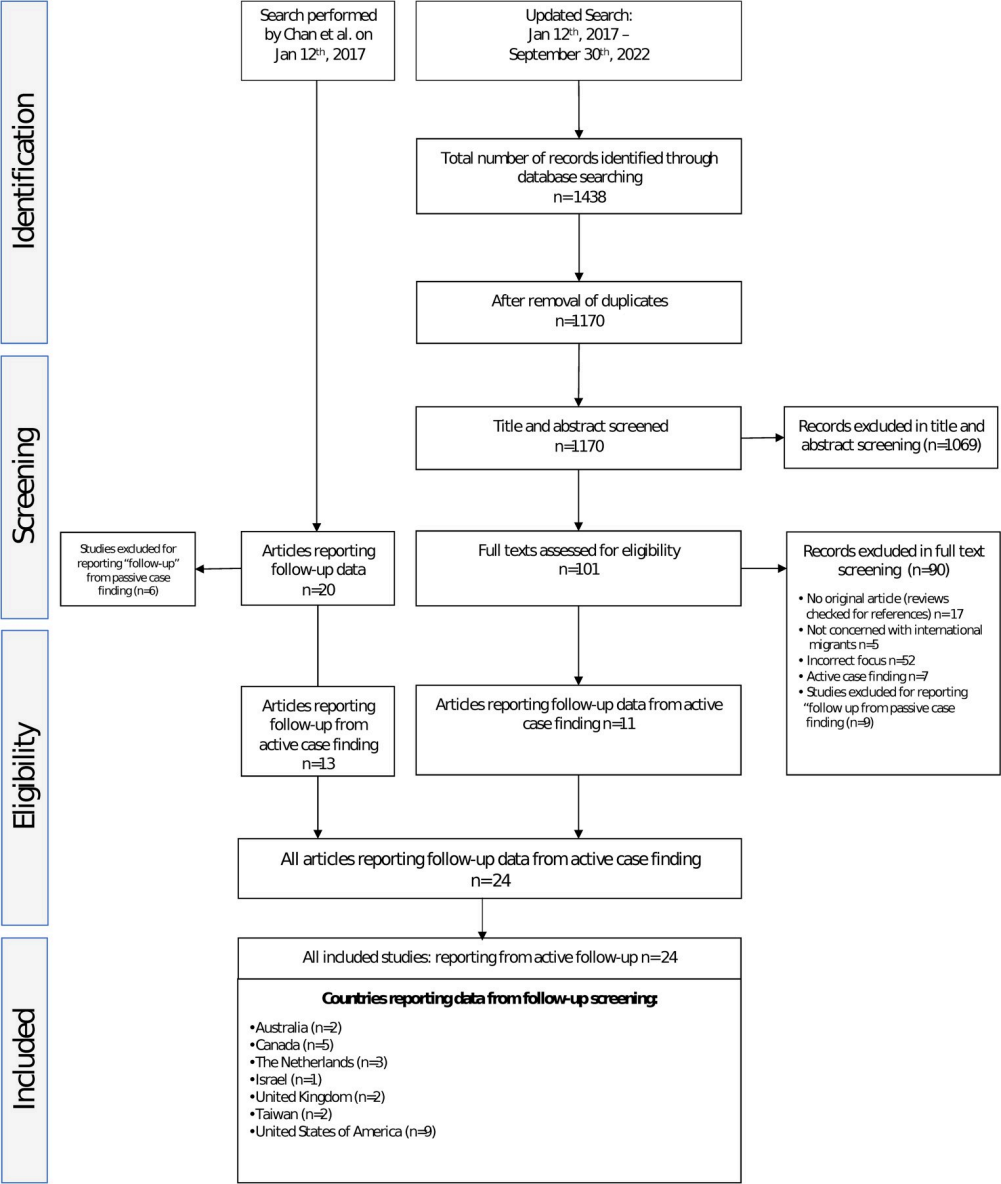
Prisma flow chart.

Of the 20 studies identified by Chan and colleagues, we included 13 that reported on active case finding. The updated literature search identified 1,170 articles after the removal of duplicates. A total of 1,069 of these were excluded through title and abstract screening and 90 through full-text screening. In total, 24 studies reporting on 26 cohorts met the inclusion criteria (see Fig 1). The included cohorts reported on programmes from 7 countries: Australia, Canada, Israel, the Netherlands, Taiwan, the United Kingdom, and the United States. We identified 1 further country, Belgium, with a follow-up screening programme; however, no reported data could be found and 1 country, Norway, with a “follow-up programme” that did not fulfil our criteria for follow-up programme (for details see annex).

The detailed review of these follow-up programmes demonstrated significant variations, in respect to the type of eligible population, legal character, and diagnostic protocols, and regarding how “follow-up” was operationalised in the context of programmes for tuberculosis prevention and control (Table 1).

**Table 1:** Key characteristics and overall design of the identified post-migration follow-up screening programmes for tuberculosis and the respective initial screening programmes.

	Initial screening		Follow-up							
Country	Screened population	Type of programme	Responsible institution	Inclusion criteria for follow-up	First contact after arrival/initial screening	Duration	Frequency	Test	Legal character	Reference
One time follow-up of pre-entry screening										
United States of America	All migrants	Pre-entry with post-entry	State/local health department	CXR abnormalities or TB history or known LTBI	Aim: 30 days, Realized: 47–53 days [23], 9 days [47]	NA	NA	Based on clinical evaluation	Voluntary	[16,21–23,25,47,48]
United Kingdom	101 countries with a high tuberculosis burden (>40 cases per 100,000 population) [35]	Pre-entry with post-entry	General practitioners	Migrants from countries with incidence ≥100/100,000 and sub-Saharan Africa [12], age 16–35 years, born in a high-incidence country (≥150 cases per 100,000 or sub-Saharan Africa) [35]	Within 5 years	NA	Once	IGRA	Voluntary	[12,19,35]
Continuous follow-up after pre-entry screening										
Israel	Ethiopian immigrants	Pre-entry with post-entry	Public health nurse	All screened	NA	One year	Continually	Clinical evaluation	Compulsory	[14]
Australia	All migrants with planned stay >6 months	Pre-entry with post-entry	Responsible state or territory body [13], local chest clinic [18]	Visa-applicants with CXR abnormalities in pre-entry screening (but negative sputum and culture results) or TB history, pregnancy, CXR lost, or poor quality, non-TB chest disease	Aim: 28 days after arrival	Length decided individually, mean: 10.3 years [18]	NA	CXR, based on clinical evaluation	Compulsory, obligation to report to a local chest clinic with 28 days after arrival [18], but not “legally enforceable”[13]	[13,18]
Canada	All migrants	Pre-entry with post-entry	Provincial health authority	CXR abnormalities or TB history	Aim: 30 days after arrival	Typically for up to 2 years [20]	NA	Clinical evaluation	Not compulsory, but a prerequisite for citizenship[20]	[24,44,49–52]
Repetitive rounds of follow-up screening of pre- and post-entry screening										
Belgium	All asylum-seekers	Post-entry	NA	“High-incidence” (no threshold reported)	Half year	One to 2 years	Half-yearly	CXR	Voluntary	[8]
The Netherlands	Migrants from country with TB incidence >50/100,000 (NNS ≤2,000) with intended stay >3 months	Post-entry	Municipal health services	All initially screened [17]; those with TB incidence ≥200/100,000 [15], countries with a high prevalence at entry screening [19]	Half year	One to 2 years [17]; 2 years [15,19]	Half-yearly	CXR	Voluntary	[8,15,17,19]
Taiwan	Migrant workers from “highly endemic”[45,53], Labour or marriage migrants from Southeast Asia or China [46]	Pre-entry and post-entry	“Centers for disease control”	Newly arrived migrants who have a verified normal CXR performed overseas	0–3 days	2.5 years	Four rounds: 0–3 days; 6, 18, and 30 months	CXR	Compulsory	[45,46,53]

CXR, chest X-ray; IGRA, interferon gamma release assay; LTBI, latent tuberculosis infection; NA, not applicable; NNS, number needed to screen; TB, tuberculosis.

The main differences were related to the number of follow-ups after initial screening (ranging from 1 to 4 rounds of screening) and time intervals (with the longest reported being a mean of 10 years in Australia), the question if programmes are voluntary and based on informed consent or mandatory, and the population subject to follow-up with some programmes being indiscriminate (i.e., following-up on all migrants initially screened) while others applied criteria to define at-risk subpopulations. Box 1 provides a contextualised overview of country approaches.

**Box 1**

Wide variations exist regarding the meaning and time frame of “follow-up.” In countries that perform pre-entry screening (e.g., US, Australia, Canada, and Israel), “follow-up” describes the first contact of a local or regional health body with the immigrant after migration [13,14,22,44]. In these countries, the follow-up refers to the time period between pre-entry screening in the country of origin and first contact in the country of destination. The first contact was not carried out in a fixed time interval, but was reported to be carried out as soon as possible after arrival (Canada: mean 61 days [44], Australia: “within 4 weeks” [13]. While some, such as the US and the UK, perform a “one-time” follow-up, and then leave it to the discretion of the medical staff to treat or follow-up on migrants with diagnosed disease or infection, other countries, such as Israel, Australia, or Canada dispose of a follow-up period, during which all previously identified migrants are followed in various visits. In Australia, the mean reported time migrants are followed is 10.3 years [18], in Canada, it is up to 2 years [20], and in Israel, a year [14] (also shown in Table 1).

In other countries, where screening is performed upon- or post-entry, such as the Netherlands and Belgium “follow-up” implies various rounds of screening for TB disease after the initial screening. These are carried out in fixed intervals over a specified interval (Netherlands and Belgium: every half year for 2 years [8,15]. Taiwan similarly performs various rounds of screening, however, disposes of a pre-entry screening, so the first round is performed 3 days after arrival, with repeat-rounds at 6 months, 18 months, and 30 months [45].

Independently of described screening algorithm, follow-up screening procedures may be voluntary, or mandatory, and even though legally binding, variations exist with respect to enforceability. Further differences were found regarding the populations screened: Whereas some countries chose to follow-up on all migrants screened initially, others only screen migrants classified as “high-risk” during the initial screening. In Australia, identification for follow-up screening takes places through the signing of “Health undertakings.” These are documents considered “legally binding, but not enforceable” [18]. In Taiwan, all 4 rounds of screening were reported to be mandatory to obtain a residency permit. Additionally, when detected with TB disease or multidrug-resistant tuberculosis (MDR) migrants may be repatriated to their countries of origin [45] (this was later changed to be only true for MDR-tuberculosis [46]). All other studies, however, do not report what legal consequences the individual may face in the case of non-compliance with the screening regulations or which measures can be taken by the responsible institution in order to ensure compliance with the screening.

### Coverage

**Fig 2:**
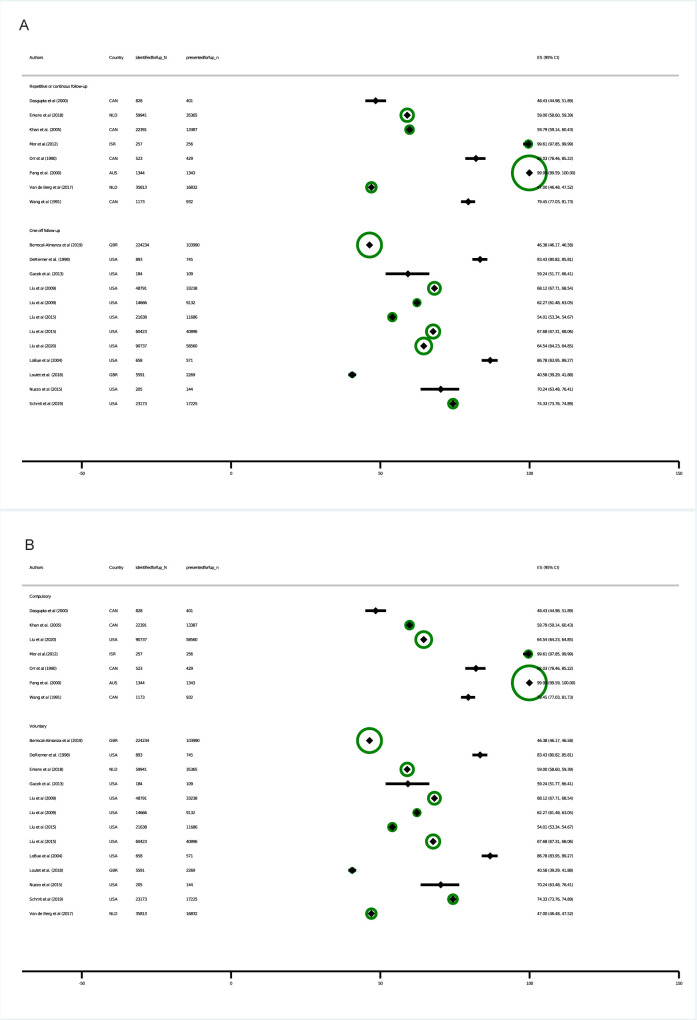
Forest plot: Coverage by follow-up (A) and legal scheme (B). Coverage: defined as the proportion of migrants who presented for screening among all migrants identified as eligible for follow-up screening. ES: estimate. Diamonds: point estimates. Horizontal bands: 95% binomial exact confidence intervals. Circles: Indicate plot weights by sample size according to the inverse of the variance. Larger circles indicate larger sample size and lower variance. Data on individuals presented for follow-up deviate in Mor and colleagues [14] and Pang and colleagues [13] by *n* = 1 from those reported in the paper to ensure that studies can be included in the plot, as studies with 100% coverage (i.e., numerator equals denominator) would otherwise be excluded.

Coverage, i.e., the proportion of migrants taking up screening among all migrants identified as eligible, was reported for 20 cohorts (see Fig 2 and Table 2). The reported coverage ranged from 41% in 1 study from the UK [12] to 100% in a study from Australia [13] and Israel [14] with 17 cohorts ranging between a coverage 45 and 85%. Overall coverage was higher than 60% in 15 studies, and tended to be lower in voluntary compared to compulsory programmes, and higher in studies from the USA, Israel and Australia.

**Table 2:** Summary of reviewed studies and outcome measures.

Authors	Year	Originally screened (N)	Identified for follow-up (n)	Identified for follow-up (%)	Presented for follow-up (first round or overall)	Coverage (%)	Cases of Tuberculosis disease (n)	Yield: Tuberculosis disease /100,000	Cases started treatment for latent tuberculosis
The Netherlands									
Van de Berg and colleagues [15]	2017	117,389	35,813	31%	16,832	47%	19	53*	··
Erkens and colleagues [17]	2008	68,122	59,941	88%	35,365	59%	47	78*	··
Boogaard and colleagues [19]	2020	26,057	26,057	100%	··	··	75	288*	··
Taiwan									
Kuan and colleagues [45]	2018	All labour migrants (on average 477,992/year) 2,248,143 screenings		100%	··	··	911	41‡	··
Kuan and colleagues [46]	2021	3,888,754 screenings		100%	··	··	2,703	70‡	··
Australia									
Kaushik and colleagues [18]	2018	··	32,550	··	··	··	84	258*	··
Pang and colleagues [13]	2000	··	1,344	··	1,344	100%	4	298*	11
Israel									
Mor and colleagues [14]	2012	13,379	257	2%	257	100%	15	5,837*	··
The United States									
DeRiemer and colleagues [47]	1998	32,054	893	3%	745	83%	39	4,367*	241
LoBue and colleagues [16]	2004	··	658	··	571	88%	39	5,927*	··
Liu and colleagues (migrants) [23]	2009	2,714,223	48,791	2%	33,238	68%	1,575	2,373*	··
Liu and colleagues (refugees) [23]	2009	378,506	14,666	4%	9,132	62%	348	3,228*	··
Gacek and colleagues [48]	2013	··	184	··	109	59%	4	2,174*	49
Liu and colleagues [22]	2015	1,650,961	21,638	1%	11,686	54%	410	1,210*	··
Liu and colleagues (diff. screening alg.) [22]	2015	1,561,460	60,423	4%	40,896	67%	731	1,895*	··
Nuzzo and colleagues [54]	2015	··	205	··	144	70%	6	2,927*	66
Liu and colleagues [21]	2020	2,102,415	90,737	4%	58,560	65%	667	735*	14,977
Schmit and colleagues. [25]	2019	··	23,173		17,225	74%	260	1,122*	··
Urban and colleagues [26]	2022	69065	69065	100%	..	..	242	350*	
Canada									
Asadi and colleagues [24]	2017	223,225	5,500	2%	··	··	39	709*	··
Orr and colleagues [49]	1990	21,586	523	2%	429	82%	19	3,633*	··
Wang and colleagues [50]	1991	21,959	1173	5%	932	79%	21	1,790*	··
Dasgupta and colleagues [51]	2000	12,898	828	6%	401	64%	4	483*	66
Khan and colleagues [20]	2015	944,375	22,391	2%	13,387	60%	102	456*	··
The United Kingdom									
Loutet and colleagues [12]	2018	··	5,591	··	2,269	41%	11	197*	449
Berrocal-Almanza and colleagues [35]	2019	485,793	224,234	46%	103,990	46%	0	0	··

* When available, yield is displayed as * active cases of tuberculosis per 100,000 identified for follow-up (eligible-population-yield)

^‡^ Alternatively, yield is displayed as ^‡^ active cases of tuberculosis per 100,000 screenings (whole-population-yield).

TB, tuberculosis.

Two studies reporting 100% coverage—or no loss to follow-up—applied obligatory screening measures, therefore, coverage of voluntary programmes tended to be lower compared with compulsory programmes (Fig 2A and 2B).

### Yields

The yields reported by the identified studies differed with respect to the denominator (see Methods, section “Coverage and yields”). Following the rationale of an intention to treat analysis, we present the “eligible-population-yield,” which was available for 24 of the 26 cohorts (the others did not report required data on the population identified as eligible for screening). Yields for the screened- and whole-population are reported in Figs A–H in S2 File.

**Fig 3:**
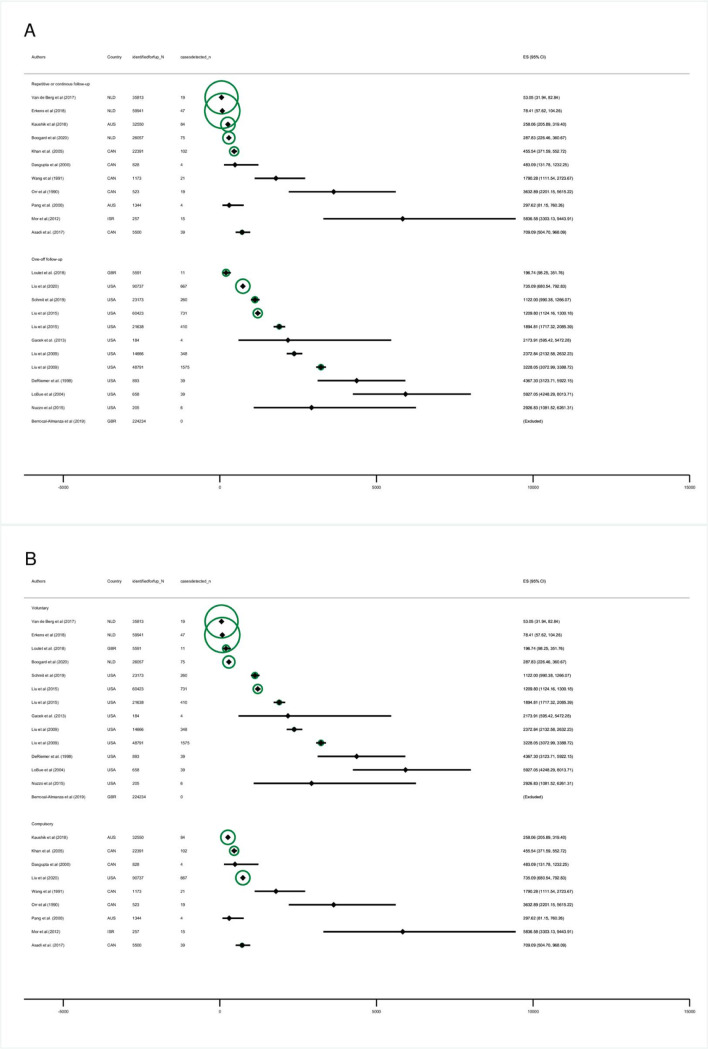
Forest plot: Eligible-population-yield by follow-up (A) and legal scheme (B). Eligible-population-yield: defined as the fraction of active TB cases with TB disease among those identified as eligible for follow-up screening, per 100,000. ES: estimate. Diamonds: point estimates. Horizontal bands: 95% binomial exact confidence intervals. Circles: Indicate plot weights by sample size according to the inverse of the variance. Larger circles indicate larger sample size and lower variance. Estimates for Berrocal-Almanza and colleagues [35] are shown as “excluded” as the numerator equals zero.

The “eligible-population-yield” of the different screening programmes varied by more than factor 100 and ranged between 53.05 (31.94 to 82.84) in a Dutch study [15] to 5,927 per 100,000 (4,248.29 to 8,013.71) in a US study [16] (see Table 2 and Fig 3). Of 15 estimates with narrow 95% CIs for yields (i.e., 95% CIs with uncertainty ranges less than 1,000 cases per 100,000) [12,13,15,17–26], 12 were below 1,500 cases per 100,000 eligible migrants (Fig 3).

**Fig 4:**
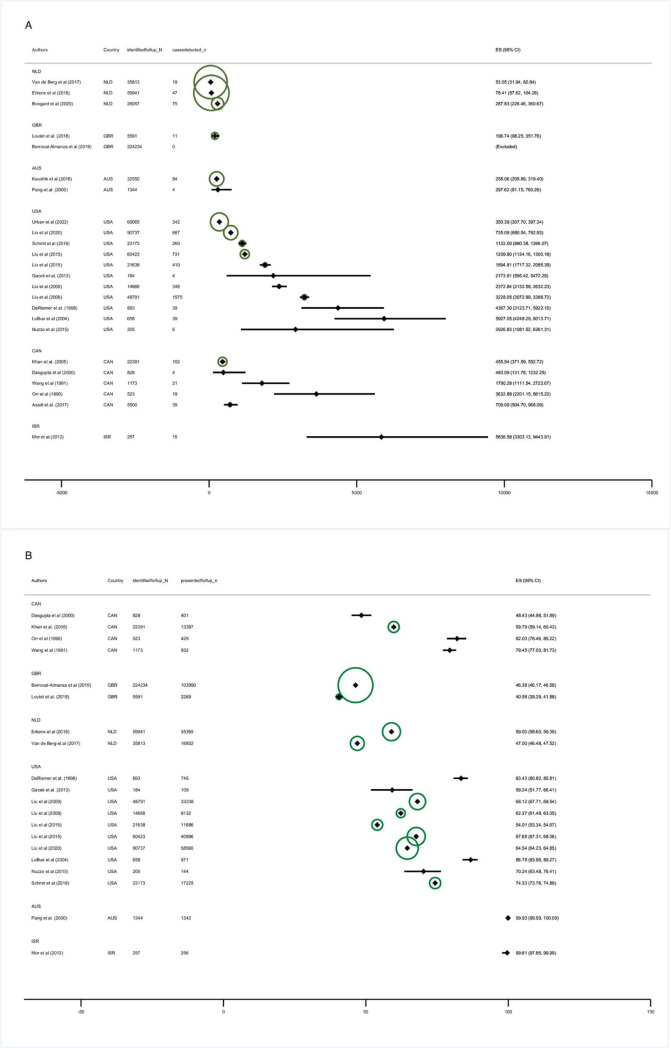
Forest plot: Eligible-population-yield (A) and Coverage (B), by country. Eligible-population-yield: defined as the fraction of active TB cases with TB disease among those identified as eligible for follow-up screening, per 100,000. Coverage: defined as the proportion of migrants who presented for screening among all migrants identified as eligible for follow-up screening. ES: estimate. Diamonds: point estimates. Horizontal bands: 95% binomial exact confidence intervals. Circles: Indicate plot weights by sample size according to the inverse of the variance. Larger circles indicate larger sample size and lower variance. Data on individuals presented for follow-up deviate in Mor and colleagues [14] and Pang and colleagues [13] by *n* = 1 from those reported in the paper to ensure that studies can be included in the plot, as studies with 100% coverage (i.e., numerator equals denominator) would otherwise be excluded. Estimates for Berrocal-Almanza and colleagues [35] are shown as “excluded” as the numerator equals zero.

One-off follow-up programmes tended to have higher yields than repetitive follow-up ones and yields in voluntary and mandatory programmes were comparable. The 2 biggest cohorts (Fig 4A) were reported from the Dutch screening programme [16,18] and showed relatively low yields (113 and 130/100,000), while some of the smaller cohorts with a highly selected study population, e.g., Israel (reporting only Ethiopian immigrants [14]) reported very high yields (5,836/100,000). However, cohorts from the same countries also varied with respect to reported yields: one study from Canada reported 455/100,000 while another reported 3,633/100,000 cases (Fig 4A).

Results of the screened- and whole-population-yields (overall and stratified by country, legal character, and follow-up scheme) are presented in Figs A–H in S2 File.

## Discussion

We systematically reviewed observational studies of post-migration follow-up programmes for prevention and control of tuberculosis with the aim to analyse and understand their design and compare the effect of programmes characteristics on coverage and yields. We found that post-migration follow-up programmes have considerable TB yields, but that voluntary screening does not perform worse than mandatory programmes. While mandatory programmes may show a slightly higher coverage of the population eligible for screening, we cannot observe patterns towards higher case finding rates. Our analysis further illustrates that existing programmes for follow-up screening differ greatly in their understanding and implementation of follow-up, including the timing and length of follow-up, the decision on whom to follow up, and the means of screening. These findings have important implications for policy makers and practitioners.

Firstly, national programmes for the prevention and control of tuberculosis may benefit significantly from going beyond a single pre- or post-entrance screening. Reactivation of latent tuberculosis infection (LTBI) contributes significantly to the burden of disease in low-incidence countries [27] and offering a post-migration follow-up screening can be an effective strategy to identify these patients early in their course of disease.

Secondly, there is a lack of evidence to support mandatory screening measures. The reported studies vary greatly in many factors, preventing a conclusion on the single effect of mandatory versus voluntary screening. Our analysis, however, shows there is no tendency that compulsory screening measures result in higher yields than voluntary measures. So far, no other study has assessed the effects of mandatory versus voluntary follow-up screening for tuberculosis, not even for the initial pre-/post-entrance screening, which is mostly conducted in a mandatory fashion [8]. Considering the significant impact of mandatory public health measures on the individuals’ rights and health autonomy [28], as well as the fact that screening for tuberculosis seems to be generally well accepted among migrants, e.g., refugee populations [29,30], this puts the ethical justifiability and rationale of this common practice into question.

Thirdly, our results highlight the need to evaluate other measures to increase programmes’ effectiveness. Coverage rates were far from 100 percent in most studies. The 2 studies reporting 100 percent coverage rates were either highly specific (Ethiopian immigrants housed in “absorption centres” for a year [14]) or geographically and organizationally distinct from the rest of the country (1 Australian state with a lean and well-organised administration-system as well as centralised care [13], Fig 4B).

This underlines the difficulties of organizing and controlling robust follow-up measures in a socioeconomically and linguistically diverse, high-mobility population. A study from the Netherlands evaluating a voluntary screening for latent infection suggested that verbal and in-person patient education, facilitating transportation, effective translation services, and personalised follow-up may increase screening uptake as well as treatment adherence and loss to follow-up [30]. Whether these measures may be more effective than mandatory screening measures has not been studied yet. But given their potential effect also on treatment adherence, which is a major barrier to effectiveness of programmes of tuberculosis control (which is not resolved by obliging people to be screened [31]), such measures should be evaluated urgently. One measure might be the combination of screening for tuberculosis with other communicable and noncommunicable diseases, as has been attempted for example in Sweden [32–34].

Finally, reporting a standard set of clinical but also meta-level data to enable programme evaluation within, but also between countries, is essential to organise effective and cost-effective public health programmes. High heterogeneity in the reported yields and a lack of reporting demographics of the screened populations as well as programmes characteristics, such as measures taken to prevent loss to follow-up, organisation of administration and screening cascade and consequences of not complying with mandatory screening measures hinders effective learning of not only what works, but also how and why.

Despite widely established pre- and post-entry screening for tuberculosis, foreign-born persons in low-incidence countries carry a high burden of disease. Offering or performing follow-up screening is just one strategy to reduce the disease prevalence. Another strategy is to prevent reactivation by screening for and offering treatment of LTBI. A recent study from the UK impressively demonstrated the potential of such a strategy: Not a single case of tuberculosis developed in the population participating in screening for latent infection [35]. Another study from the Netherlands showed that half of the foreign-born population had been in the country for longer than 5 years at the time of diagnosis and could not have been detected through the existing follow-up approach. However, a good proportion could have likely been prevented by (latent) TBI screening and treatment [30].

Therefore, combining screening for TB disease and TBI, combined with TB symptom screening, could be a reasonable approach as TB disease must be ruled out for TBI diagnosis and serological or skin testing have been suggested to be valid methods for screening for TB disease [36]. TBI screening further has the potential to identify cases detected later through self-reporting, increasing potential effectiveness and cost effectiveness beyond the yields reported for follow-up screening.

Furthermore, screening for TB disease is usually implemented via chest X-ray and can therefore only identify pulmonary tuberculosis. This may be sensible from a public health perspective, as usually only pulmonary disease is considered infectious. From an individual health perspective, however, non-pulmonary disease constitutes a relevant burden of disease (e.g., a third of all cases of TB disease in foreigners in Germany [37]) and may be at least as detrimental to the individuals’ health. Screening for TBI has the benefit of potentially detecting extrapulmonary disease, as was demonstrated by a recent study from the Netherlands [36].

Regarding cost-effectiveness, evidence from Germany points to lower costs per case prevented by screening for LTBI [38] than the current indiscriminate and mandatory upon-entry screening for TB disease [39]. Further economic modelling and intervention studies are needed to decide if or when follow-up screening or upon-entry screening for latent infection may be more effective, cost-effective and accepted in which setting and for which populations. In line with the recent call for reciprocity by Beeres and colleagues [28], more comprehensive health care measures, including screening for latent, pulmonary and non-pulmonary tuberculosis, as well as expanding to providing high-quality, accessible care for noncommunicable disease might increase acceptance and coverage of screening programmes and benefit migrants and host-populations alike. Future studies should also perform qualitative and cross-nationally comparative studies on relevant aspects of the design and implementation of follow-up programmes.

The identified screening programmes were highly heterogenous in their design. Despite stratifying for essential programmes characteristics, heterogeneity was high in the reported outcome measures (I^2^ > 92% for all chosen parameters). This is likely due to the multitude of factors that contribute to the effectiveness of a screening programme: Among others, these include patient education, programme management, and training, as well as administrative procedures. Information on these aspects were scant and widely absent in existing reports. The number of migrants that may have left the country before qualifying for follow-up screening was not reported and may have affected the overall effectiveness.

Furthermore, our analysis used a binary classification of mandatory and voluntary based on the legal background described in the respective studies. The reality might be more complex. Whether participation in a voluntary screening measure is in fact perceived as mandatory may be influenced by the legal consequences of refusing to participate and whether the voluntary nature of measures is adequately explained (e.g., using interpreters) and perceived as such.

Additionally, screening effectiveness depends greatly on the baseline incidence of the disease, i.e., the prevalence of LTBI and its reactivation rate. The individual risk for both has been shown to vary greatly depending on country of origin, travel route, age, sex, health, social status, and reason for migration [40–43]—to only mention a few. Detailed data on the screened population with respect to these factors was widely absent in most studies, not allowing for a more detailed stratification. Additionally, some countries performed follow-up screening on all migrants screened initially, whereas other countries followed up on those migrants considered to be “at high-risk,” however differing in their understanding of high-risk (e.g., abnormal finding on initial screening, a history of tuberculosis, poor quality of chest X-ray) and on the percentage of initially screened persons this includes. These aspects may have also affected the results of the comparison between one-off and repetitive screening measures. For example, the Netherlands offer repetitive follow-up via voluntary, biannual chest X-rays, based on the incidence of country of origin without further preselection while other countries in the one-off group defined the population “at risk” more narrowly.

Despite the inherent heterogeneity in our estimates, to our knowledge our analysis is the first to assess the effects of differences in screening design on the effectiveness of screening and therefore substantially contributes to the scarce evidence on how to design effective post-migration screening programmes for tuberculosis disease.

The programme characteristics of post-migration follow-up screening for prevention and control of tuberculosis considerably affect coverage and yield. Voluntary screening seems to achieve similar yields as mandatory screening measures, putting into question the ethical justifiability and rationale of this common practice. Mandatory programmes achieve higher coverage, but this benefit alone may not outweigh human rights and ethical considerations of voluntary programmes.

Heterogeneity between the existing programmes is tremendous and can be of use for learning how to design rational, efficient, and cost-efficient follow-up screening programmes. However, divergent outcome parameters, underreporting of relevant programmes characteristics, and demographics of the study population hinder effective learning and knowledge transfer. Efforts are urgently needed to harmonise the reporting of outcome parameters, process design and qualitative elements to optimise coverage and screening cascades. Furthermore, the value of follow-up screening needs to be evaluated as an individual measure in the toolbox of possible strategies for the prevention and control of tuberculosis, comparing its costs, effectiveness, and acceptability for the individuals affected to other possible measures such as a (targeted) initial screening, screening, and offering treatment for latent disease and strategies to enabling better and earlier passive case finding and improving access to care.

## Supporting information

S1 File Prisma check list. (DOCX)

S2 File Table A: Search strategy; Table B: Inclusion/Exclusion criteria; Table C: Summary of meta-analytic results obtained from Random Effect Models; Figs A–H: Forest plots of yields; Figs I–T: Forest plots of pooled estimates.
(DOCX)

## Abbreviations

CI: confidence interval
LTBI: latent tuberculosis infection
MDR: multidrug-resistant
TBI: tuberculosis infection
