# Supplementary material for: Mandatory, voluntary, repetitive, or one-off post-migration follow-up for tuberculosis prevention and control: A systematic review
Source: PLoS Med. 2023 Jan 31;20(1):e1004030. doi: 10.1371/journal.pmed.1004030 (PMC9888720; doi:10.1371/journal.pmed.1004030)
Supplement: S2 File — Table A: Search strategy; Table B: Inclusion/Exclusion criteria; Table C: Summary of meta-analytic results obtained from Random Effect Models; Figs A–H: Forest plots of yields; Figs I–T: Forest plots of pooled estimates. (DOCX) [file pmed.1004030.s002.docx]

A systematic review comparing mandatory, voluntary, repetitive or one-off post-migration follow-up for tuberculosis prevention and control

S2 File

Contents

1. Glossary of terms
2. Systematic review
   1. Detailed Search strategy *(Table A)*
   2. Inclusion and Exclusion Criteria
3. Meta-analysis
   1. Methodology
   2. Results
4. Figures and Tables
   1. Inclusion/Exclusion criteria (T*able B*)
   2. Summary of meta-analytic results (T*able C*)
   3. Forest plots without pooled estimates for
      1. ‘Screened-population-yield’ (*Figs A-D)*
      2. ‘Whole-population-yield (*Figs E-H)*
   4. Forest plots with pooled estimates for
      1. Coverage (*Figs I-K)*
      2. ‘Screened-population-yield’ (*Figs L-N*)
      3. ‘Eligible-population-yield (*Figs O-Q*)
      4. ‘Whole population yield (*Figs R-T*)
5. References

1 Glossary of Terms

**Pre-entry screening:** Screening of persons who intend to migrate, usually before they leave their home country [1].

**Post-entry screening:** Screening performed after arrival at the destination country, either immediately or after some (defined or undefined) time-lag.

**Upon-entry:** Generally associated with screening immediately at borders or airports. As the distinction between upon-entry and post-entry screening is vague and pre-defined or legal requirements may deviate from real life implementation, we decided against differentiating between post- und upon-entry screening. We hence refer to any screening performed after arrival at the destination country as post-entry screening, regardless of the time interval between crossing borders or the location of screening.

**Follow-up screening:** The term is understood differently by countries and included primary studies (see Table 1, main manuscript). For inclusion in this review, we consolidated the different meanings and considered “follow-up screening” as all programmes which:

- Performed an intial round of screening (either pre-, or post/-upon-entry), and
- Obtained a final conclusion/result of the initial round to conclude on prevalent TB disease (“positive”, “negative”, “negative but at risk” due some criterion), and
- Performed one or more rounds of screening (among those who were “negative” or “negative but at risk” *after* concluding on the results of the first round).

2 Systematic review

**Search strategy**

Database: Ovid MEDLINE January 11^th^, 2017 until November to September 30th, 2022 and Embase Classic+Embase

Search Strategy (as conducted by Chan et al, [2]).

**Table A**

| 1 | Tuberculosis/ or TB.mp. |
| --- | --- |
| 2 | immigrants.mp. or "Emigrants and Immigrants" |
| 3 | migrants.mp. or "Transients and Migrants"/ |
| 4 | ((over$seas or foreign) adj3 born).mp. [mp=title, abstract, original title, name of substance word, subject heading word, keyword heading word, protocol supplementary concept word, rare disease supplementary concept word, unique identifier] |
| 5 | (new adj3 (arrival* or entrant*)).mp. [mp=title, abstract, original title, name of substance word, subject heading word, keyword heading word, protocol supplementary concept word, rare disease supplementary concept word, unique identifier] |
| 6 | (Visa adj3 appl*).mp. [mp=title, abstract, original title, name of substance word, subject heading word, keyword heading word, protocol supplementary concept word, rare disease supplementary concept word, unique identifier] |
| 7 | Refugees/ or refugee*.mp. |
| 8 | asylum adj3 seek*).mp. [mp=title, abstract, original title, name of substance word, subject heading word, keyword heading word, protocol supplementary concept word, rare disease supplementary concept word, unique identifier] |
| 9 | screening.mp. |
| 10 | Surveillance.mp. or Public Health Surveillance/ |
| 11 | follow*up.mp. |
| 12 | Health undertaking.mp. |
| 13 | 2 or 3 or 4 or 5 or 6 or 7 or 8 |
| 14 | 9 or 10 or 11 or 12 |
| 15 | 1 and 13 and 14 |

**Inclusion and exclusion criteria**

Inclusion and exclusion criteria are shown in Table B.

Explanation for the exclusion of Harstadt *et a l*[ 3]**:** Harstadt et al is a study that uses the term “follow-up” in context of a screening programme for Tuberculosis and had been included by Chan et al as a follow-up screening. However, when looking into detail in the screening programme of Harstadt et al., it differs from other “follow-up”-programmes as it is understood as “follow-up diagnostics” or “work-up” to the initial post-entry screening: E.g., further diagnostic tests for a person who in the initial chest-x-ray had a pathology and in whom TB disease had not been ruled out initially. This is in line with the relatively short follow-up times (9-10 weeks) -in contrast to the other screening programmes which are characterised by longer periods between first completed screening and a second diagnostic test.

3 Meta-analyses

**Methodology**

We calculated coverage as the proportion of migrants who presented for screening among all migrants identified as eligible for follow-up screening. As the definition of yield reported by the identified studies varied, we recalculated yields based on three different denominators to quantify the cascade of screening. Yields were defined as the fraction of active TB cases among (i) those presented for follow-up screening (‘screened-population-yield’), (ii) those identified as eligible for follow-up screening (‘eligible-population-yield’), and (iii) the total population initially screened for eligibility (‘whole-population-yield’).

We tabulated key-characteristics and provided a narrative synthesis of follow-up programs. We then pooled the reported coverage and yields with different denominators (screened-/eligible-/and whole-population-yields), stratified by program characteristic focusing on the legal character of programs (voluntary vs. compulsory) and the scheme of follow-up screening (one-off vs. repetitive rounds or continuous follow-up). We used random effects models with the Freeman and Tukey double arcsine transformation to obtain pooled estimates and 95% confidence intervals (CI) using the ‘metaprop’ command in StataSE 15 [4]. We used the I^2^ statistic to quantify heterogeneity, defined as the percentage of the variability in effect estimates that is due to heterogeneity rather than sampling error [5], and tested for heterogeneity between subgroups by means of chi-2 tests (testing the null-hypothesis of no difference in heterogeneity between subgroups).

**Results of the meta-analyses**

The overall pooled estimate for coverage across studies was 70·27% (95% CI: 65·39 – 74·92). We found significant (p< 0·001) heterogeneity between subgroups: coverage in voluntary programs was about 18.2 percentage-points lower compared to compulsory programs, and programs with one-off follow-up schemes had 12·0 percentage-points lower uptake than programs with continuous or repetitive follow-up.

Results of the meta-analysis of the coverage are displayed in Fig I (overall), Fig J (stratified by programme design) and Fig K (stratified by legal schema).

The pooled overall ‘eligible-population-yield’ estimate was 1,183 per 100,000 with significant heterogeneity (I2=99·75%). The program design had a significant effect on yield (see Table C, below) and one-off follow-up programs reported higher yields than continuous or repetitive follow-up schemes. Legal character of the program did not have a significant effect on yields. Results of the meta-analysis of ‘screened-population-yield’ (yield 1) are shown in Figs L-N (overall, stratified by programme design and legal schema) and D. Results of ‘eligible-population-yield’ (yield 2) are shown in figure O-Q and results of ‘whole-population-yield’ (yield 3) are shown in Figs Q-T.

An overview of all pooled results, performed as exploration, are presented in Table C. Figs A-D show forest plots of ‘screened population yield’ (yield 1) without pooled estimates (overall, stratified by country, by programme character and legal schema) while Figs E-H show the same for ‘whole-population-yield (yield 3). The respective figures for ‘eligible-population-yield (yield 2) and coverage are reported in the main manuscript.

4 Further figures and Tables

**Table B: Inclusion/Exclusion criteria**

|  | **Inclusion criteria** | **Exclusion criteria** | **CODE for exclusion** |
| --- | --- | --- | --- |
| Type of population | Not included in IOM definition of international migrant ("any person who is moving or has moved across an international border or within a state away from his/her habitual place of residence") + ONLY ADULTS | Not complying with type of population | 1 |
| Type of study | Published original articles and reviews | Not complying with type of articles | 2 |
| Focus of study | Focus of study: Studies which evaluate the rate of tuberculosis post-migration in international migrants, who had previously been screened for active tuberculosis in premigration screening programs (by any method), this screening had been concluded with no active TB or at risk for TB (see also definition of follow-up screening in glossary) | Not complying with focus of study | 3 |
| Reported outcome | Cumulative incidence rate or rate over time, detected by follow-up (involving in-person review of high-risk migrants with pre-migration screening abnormalities) | Not complying with outcome measures | 4 |
| Mode of case finding | Active case finding | Passive case finding | 5 |

**Table C*:* Summary of meta-analytic results obtained from Random Effect Models**

|  | **Random Pooled  Estimate (95% CI)** | **No of studies** | **Variation in estimate  attributable to heterogeneity (I2)** | **Heterogeneity  chi2 (df)** | **p-value** | **Tau^2** | **z-statistic  (Test of ES=0)** | **p-value** | **Test for heterogeneity  between sub‐groups** |
| --- | --- | --- | --- | --- | --- | --- | --- | --- | --- |
| **Coverage** |  |  |  |  |  |  |  |  |  |
| Overall | **70.27 (65.39 - 74.92)** | 20 | **99,93%** | 27709.72 (19) | **<0.001** | 0,06 | 36,7 | **<0.001** |  |
| By legal character |  |  |  |  |  |  |  |  |  |
| *Voluntary screening* | **63.53 (57.13 - 69.70)** | 13 | **99,94%** | 21081.12 (12) | **<0.001** |  | 27,17 | **<0.001** |  |
| *Compulsory screening* | **81.68 (73.46 - 88.67)** | 7 | **99,80%** | 2992.45 (6) | **<0.001** |  | 22,03 | **<0.001** | **<0.001** |
| By follow-up scheme |  |  |  |  |  |  |  |  |  |
| *One-time follow-up* | **65.36 (58.40 - 72.00)** | 12 | **99,95%** | 22508.11 (11) | **<0.001** |  | 25,32 | **<0.001** |  |
| *Repetitive rounds  or Continuous follow-up* | **77.26 (69.30 - 84.33)** | 8 | **99,87%** | 5201.23 (7) | **<0.001** |  | 23,02 | **<0.001** | **0,02** |
| **Yield 1** |  |  |  |  |  |  |  |  |  |
| Overall | **1924.72 (1037.17 - 3064.76)** | 20 | **99,75%** | 7605.74 (19) | **<0.001** | 0,03 | 6,61 | **<0.001** |  |
| By legal character |  |  |  |  |  |  |  |  |  |
| *Voluntary screening* | 2021.95 (806.03 - 3743.20) | 13 | **99,84%** | 7374.47 (12) | **<0.001** |  | 4,8 | **<0.001** |  |
| *Compulsory screening* | 1484.14 (969.70 - 2099.71) | 7 | **92,39%** | 78.89 (6) | **<0.001** |  | 9,08 | **<0.001** | 0,48 |
| By follow-up scheme |  |  |  |  |  |  |  |  |  |
| *One-time follow-up* | 2487.82 (1103.48 - 4383.89) | 12 | **99,84%** | 6953.87 (11) | **<0.001** |  | 5,31 | **<0.001** |  |
| *Repetitive rounds  or Continuous follow-up* | 1046.13 (534.13 - 1713.71) | 8 | **97,19%** | 249.26 (7) | **<0.001** |  | 6,08 | **<0.001** | 0,07 |
| **Yield 2** |  |  |  |  |  |  |  |  |  |
| Overall | **1183.75 (675.07 - 1825.42)** | 23 | **99,75%** | 8830.40 (22) | **<0.001** | 0,02 | 7,18 | **<0.001** |  |
| By legal character |  |  |  |  |  |  |  |  |  |
| *Voluntary screening* | 1258.11 (528.69 - 2277.51) | 14 | **99,84%** | 8376.71 (13) | **<0.001** |  | 5,03 | **<0.001** |  |
| *Compulsory screening* | 866.54 (567.86 - 1223.65) | 9 | **96,05%** | 202.69 (8) | **<0.001** |  | 9,11 | **<0.001** | 0,39 |
| By follow-up scheme |  |  |  |  |  |  |  |  |  |
| *One-time follow-up* | **1700.28 (736.84 - 3034.09)** | 12 | **99,87%** | 8215.57 (11) | **<0.001** |  | 5,2 | **<0.001** |  |
| *Repetitive rounds  or Continuous follow-up* | **559.69 (339.38 - 830.26)** | 11 | **97,22%** | 359.21 (10) | **<0.001** |  | 7,79 | **<0.001** | **0,02** |
| **Yield 3** |  |  |  |  |  |  |  |  |  |
| Overall | **51.38 (35.89 - 69.57)** | 16 | **99,16%** | 1790.07 (15) | **<0.001** | 0 | 10,97 | **<0.001** |  |
| By legal character |  |  |  |  |  |  |  |  |  |
| *Voluntary screening* | 57.53 (34.33 - 86.61) | 9 | **99,42%** | 1387.72 (8) | **<0.001** |  | 8,11 | **<0.001** |  |
| *Compulsory screening* | 41.79 (25.20 - 62.34) | 7 | **97,09%** | 205.86 (6) | **<0.001** |  | 7,87 | **<0.001** | 0,37 |
| By follow-up scheme |  |  |  |  |  |  |  |  |  |
| *One-time follow-up* | 41.72 (23.59 - 64.93) | 7 | **99,53%** | 1267.46 (6) | **<0.001** |  | 7,51 | **<0.001** |  |
| *Repetitive rounds  or Continuous follow-up* | 62.55 (32.34 - 102.29) | 9 | **97,60%** | 332.66 (8) | **<0.001** |  | 6,36 | **<0.001** | 0,27 |

Coverage: defined as the fraction of those presented for screening among all individuals identified as eligible for follow-up screening. Yield 1: the fraction of active TB cases among those presented for follow-up screening. Yield 2: the fraction of active TB cases among those identified as eligible for follow-up screening. Yield 3: the fraction of active TB cases among the total population initially screened for eligibility. Chi2: chi-square test. Df: degrees of freedom.


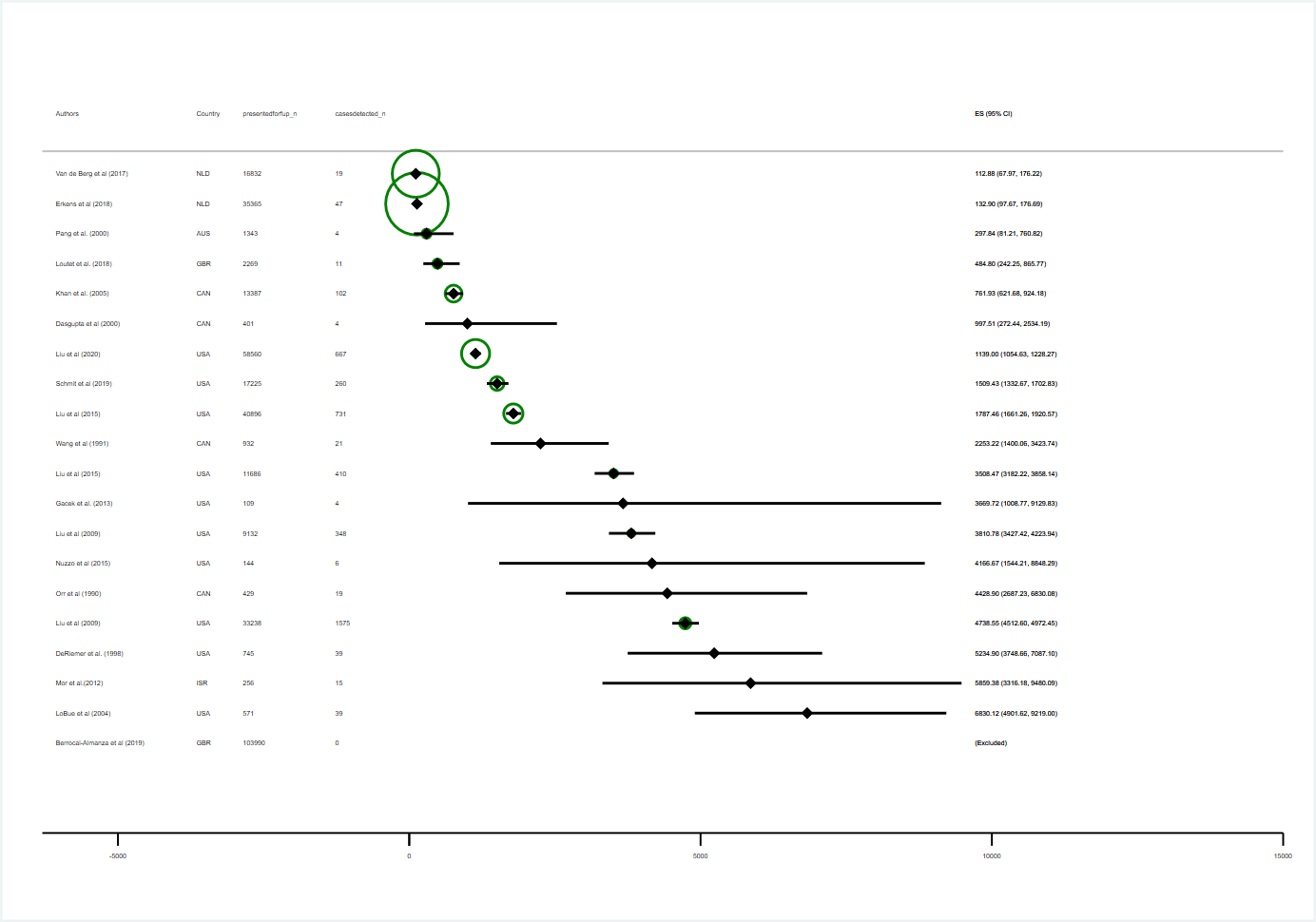
**Fig A: Forest plot of ‘screened-population-yield’**

ES: estimate of yield, per 100.000. Diamonds: point estimates. Horizontal bands: 95 % binomial exact confidence intervals. Circles: Indicate plot weights by sample size according to the inverse of the variance. Larger circles indicate larger sample size and lower variance. Estimates for Berrocal-Almanza et al. are shown as “excluded” as the numerator equals zero.

Diamonds: point estimates. Horizontal bands: 95 % binomial exact confidence intervals. Circles: Indicate plot weights by sample size according to the inverse of the variance. Larger circles indicate larger sample size and lower variance


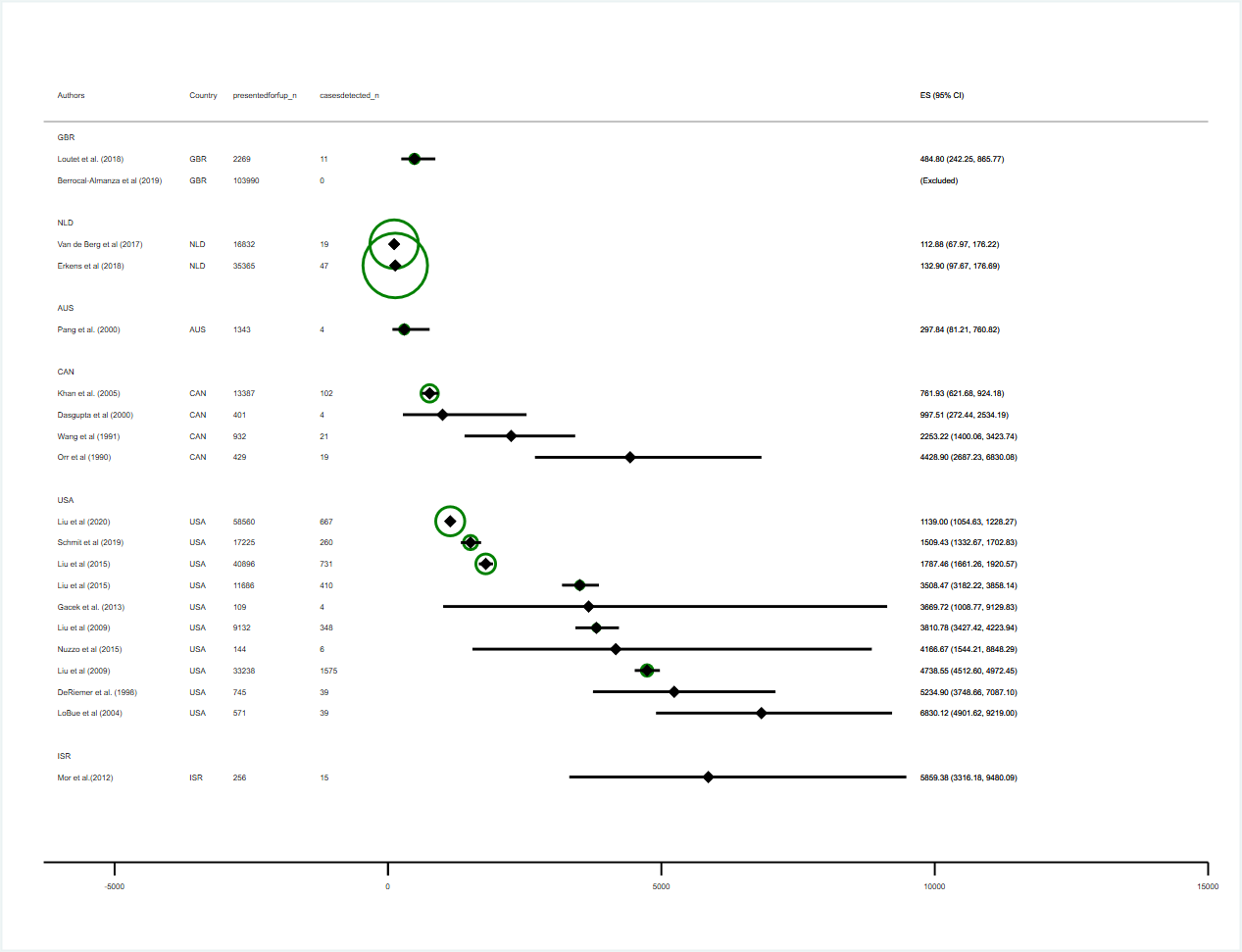


ES: estimate of yield, per 100.000. Diamonds: point estimates. Horizontal bands: 95 % binomial exact confidence intervals. Circles: Indicate plot weights by sample size according to the inverse of the variance. Larger circles indicate larger sample size and lower variance. Estimates for Berrocal-Almanza et al. are shown as “excluded” as the numerator equals zero.

**Fig B: Forest plot of ‘screened-population-yield’, stratified by country**


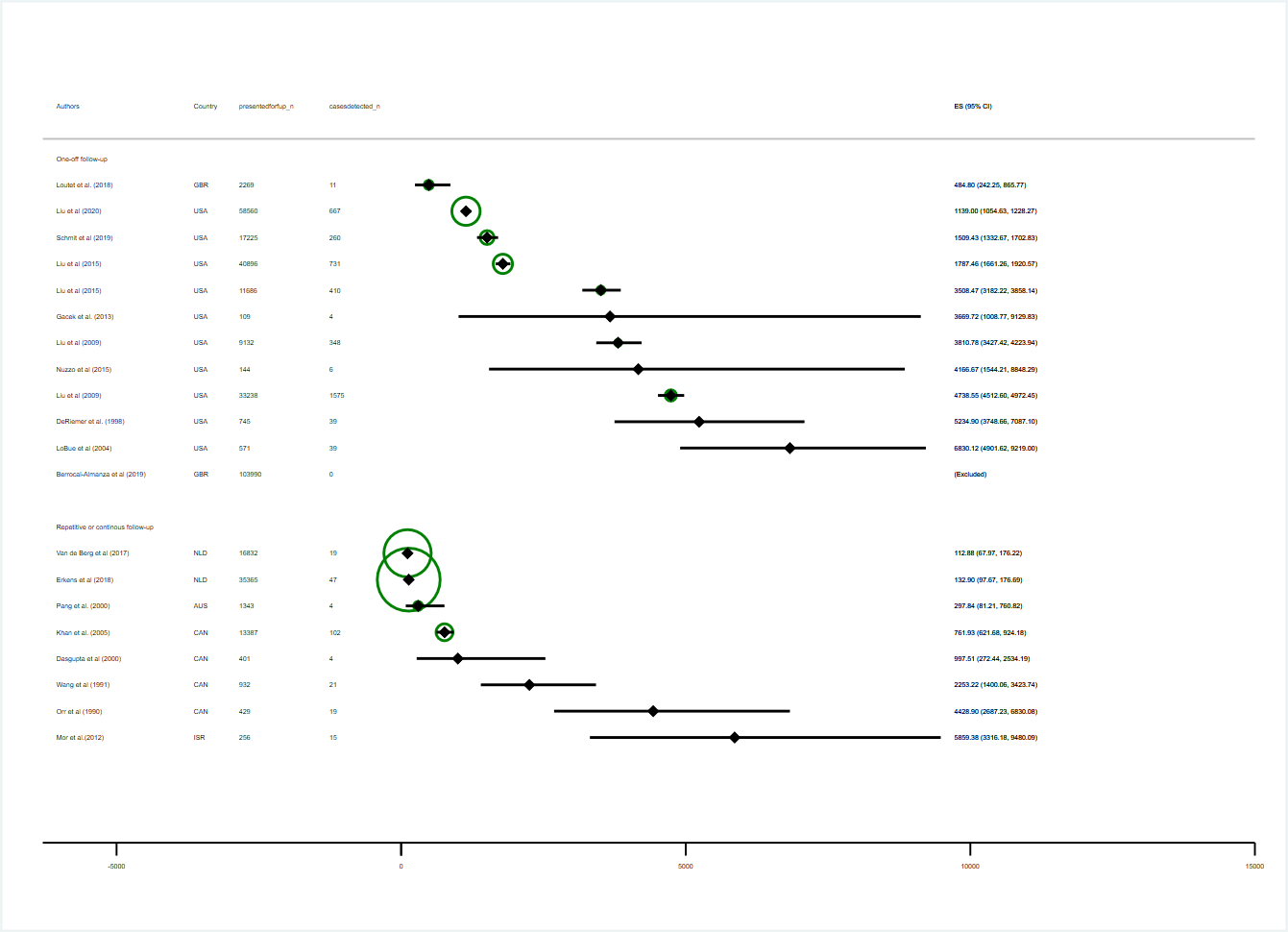
**Fig C: Forest plot of ‘screened-population-yield’, stratified by programme design**

ES: estimate of yield, per 100.000. Diamonds: point estimates. Horizontal bands: 95 % binomial exact confidence intervals. Circles: Indicate plot weights by sample size according to the inverse of the variance. Larger circles indicate larger sample size and lower variance. Estimates for Berrocal-Almanza et al. are shown as “excluded” as the numerator equals zero.


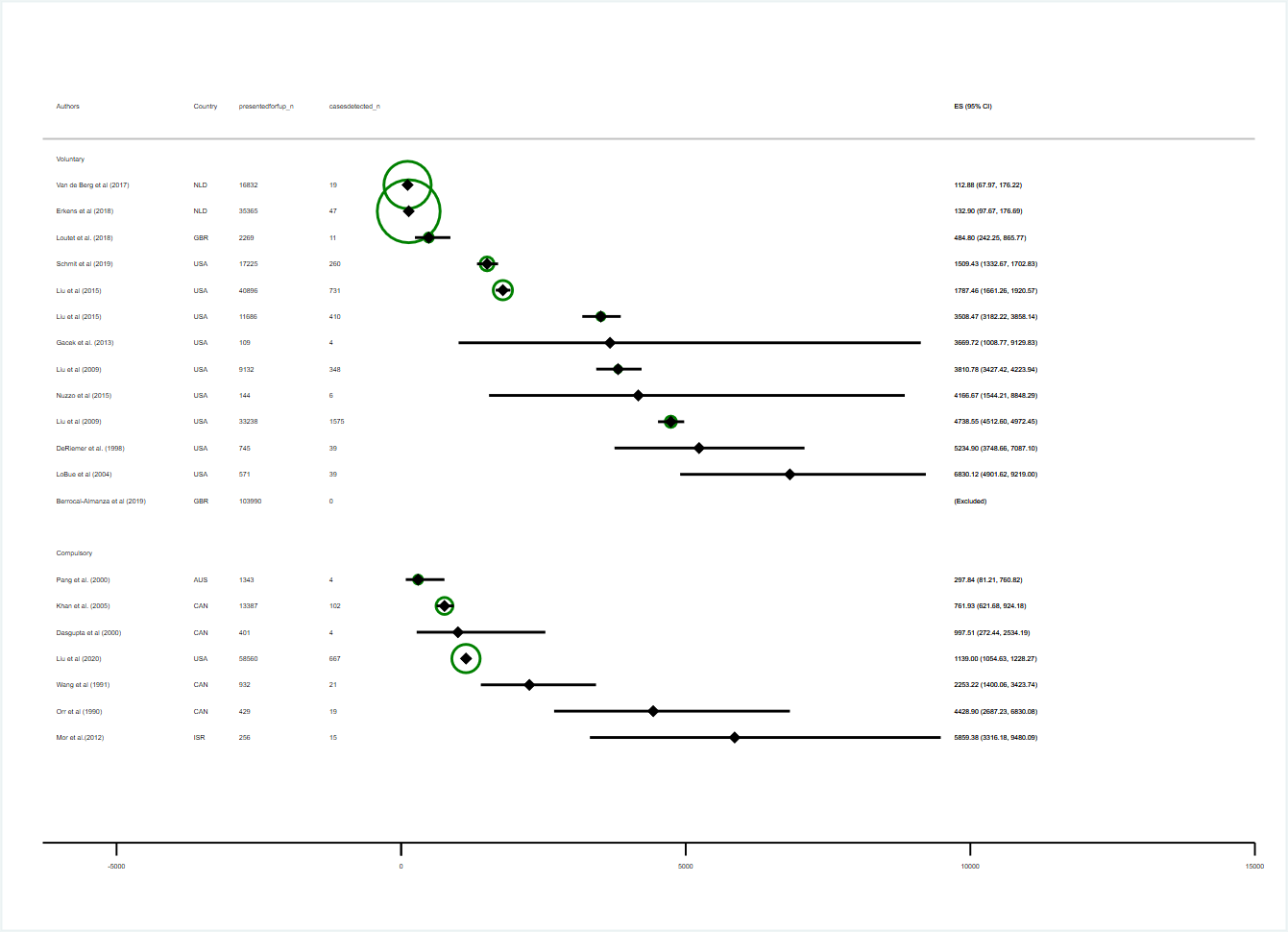
**Fig D: Forest plot of ‘screened-population-yield’, stratified by legal schema**

ES: estimate of yield, per 100.000. Diamonds: point estimates. Horizontal bands: 95 % binomial exact confidence intervals. Circles: Indicate plot weights by sample size according to the inverse of the variance. Larger circles indicate larger sample size and lower variance. Estimates for Berrocal-Almanza et al. are shown as “excluded” as the numerator equals zero.


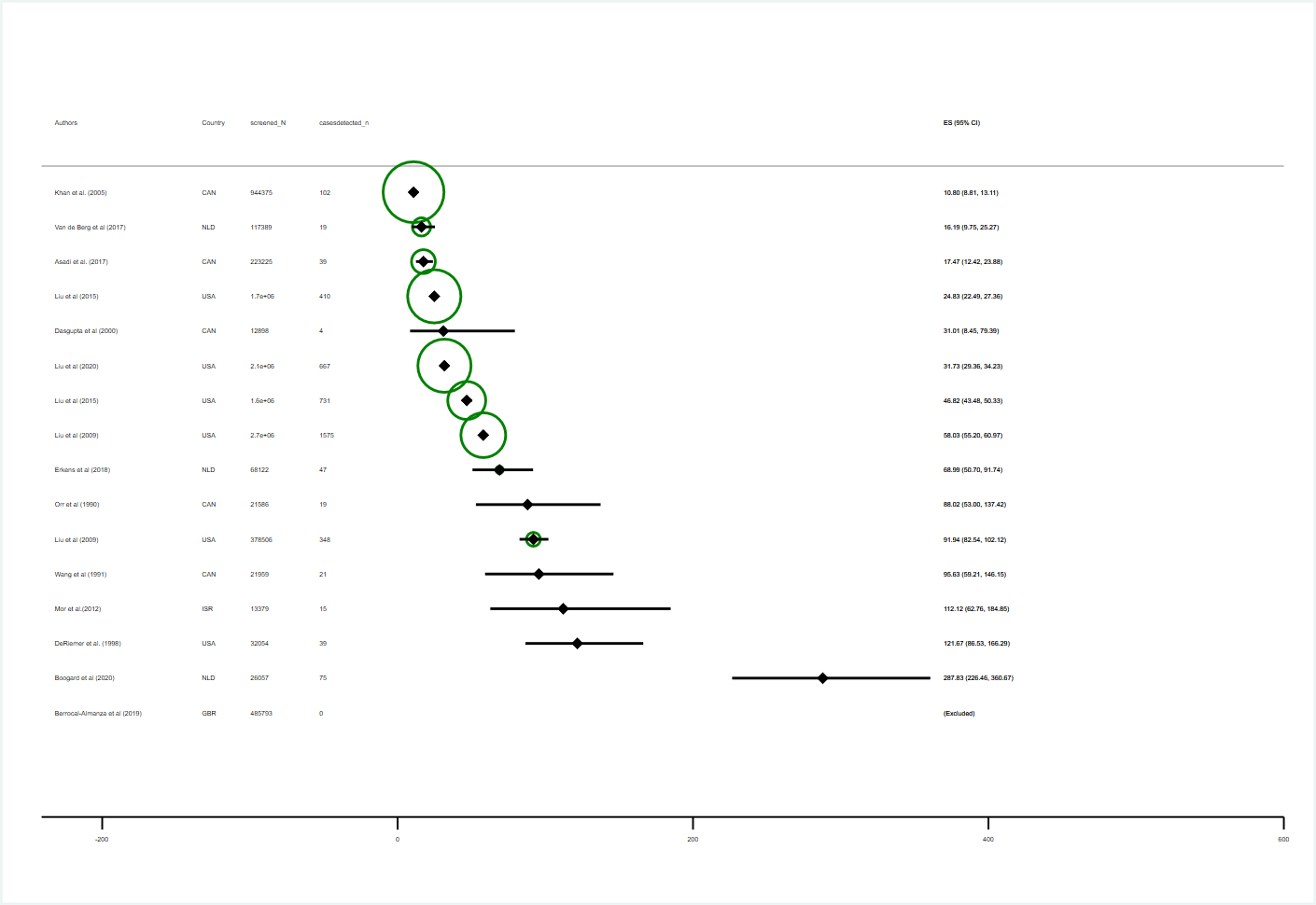


ES: estimate of yield, per 100.000. Diamonds: point estimates. Horizontal bands: 95 % binomial exact confidence intervals. Circles: Indicate plot weights by sample size according to the inverse of the variance. Larger circles indicate larger sample size and lower variance. Estimates for Berrocal-Almanza et al. are shown as “excluded” as the numerator equals zero.

**Fig E: Forest plot of ‘whole-population-yield’**

**
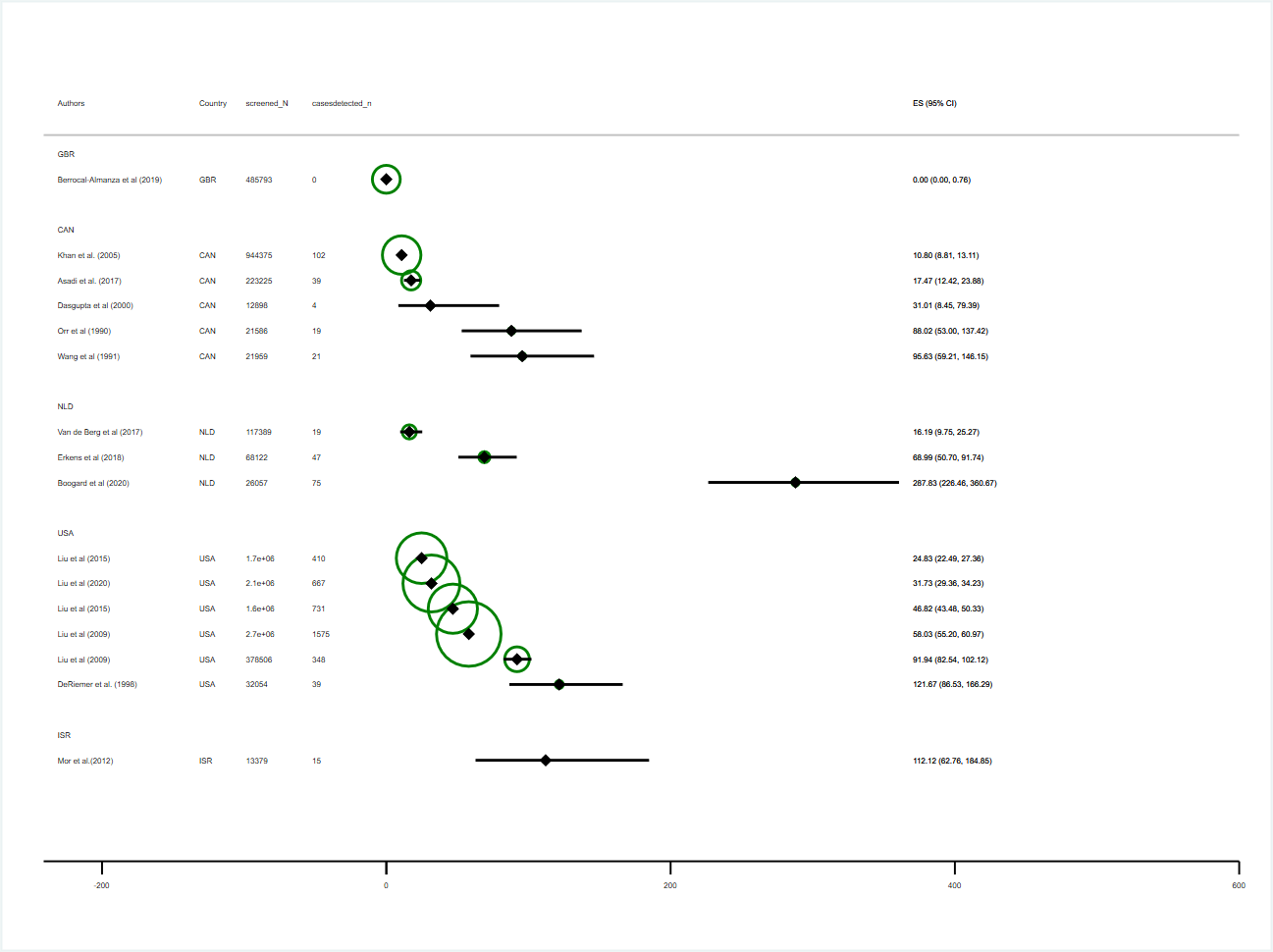
**

**Fig F: Forest plot of ‘whole-population-yield’, stratified by country**

ES: estimate of yield, per 100.000. Diamonds: point estimates. Horizontal bands: 95 % binomial exact confidence intervals. Circles: Indicate plot weights by sample size according to the inverse of the variance. Larger circles indicate larger sample size and lower variance

**
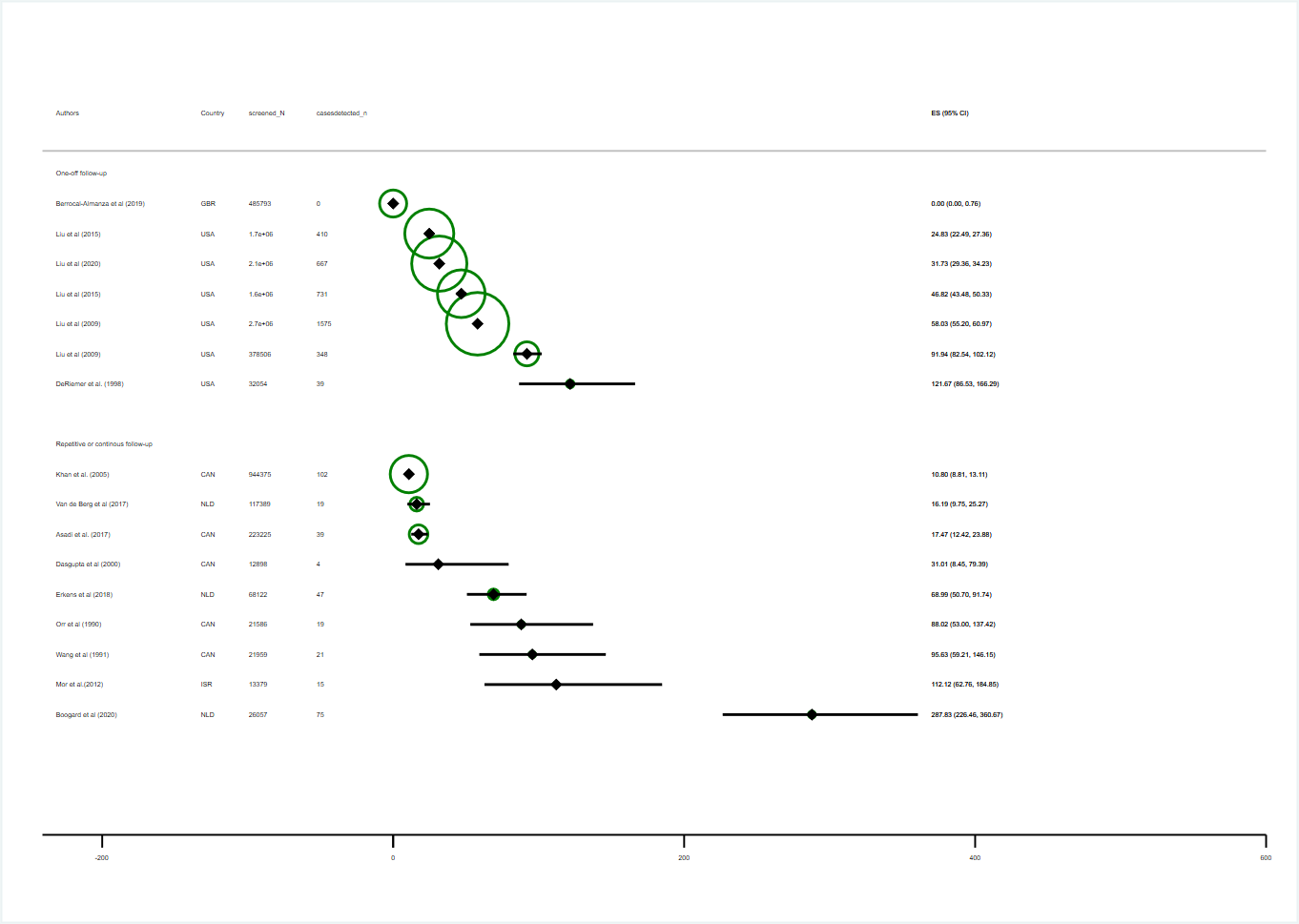
**

ES: estimate of yield, per 100.000. Diamonds: point estimates. Horizontal bands: 95 % binomial exact confidence intervals. Circles: Indicate plot weights by sample size according to the inverse of the variance. Larger circles indicate larger sample size and lower variance

**Fig G: Forest plot of ‘whole-population-yield’, stratified by programme design**

**
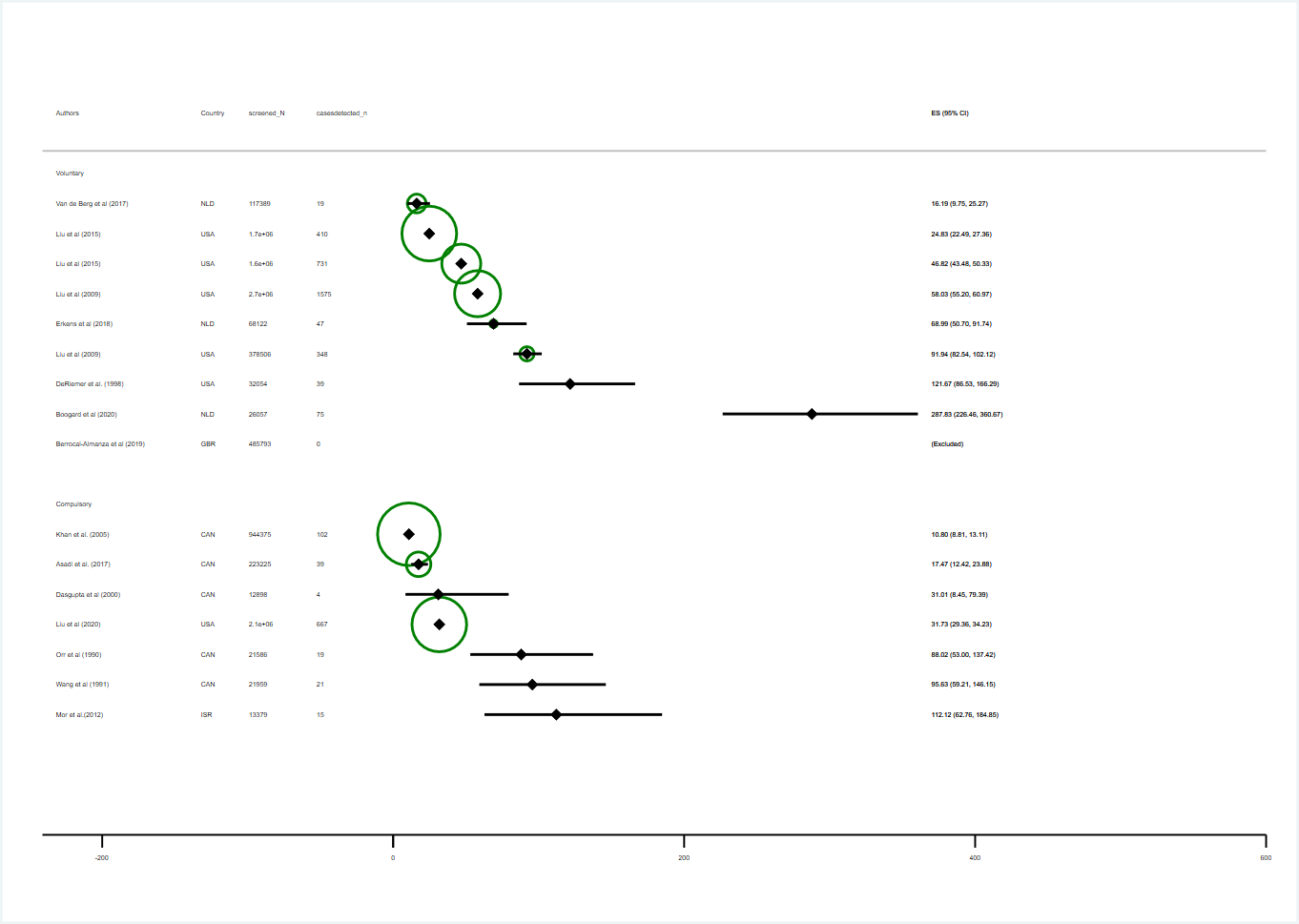
**

ES: estimate of yield, per 100.000. Diamonds: point estimates. Horizontal bands: 95 % binomial exact confidence intervals. Circles: Indicate plot weights by sample size according to the inverse of the variance. Larger circles indicate larger sample size and lower variance. Estimates for Berrocal-Almanza et al. are shown as “excluded” as the numerator equals zero.

**Fig H: Forest plot of ‘whole-population-yield’, stratified by legal schema**

**
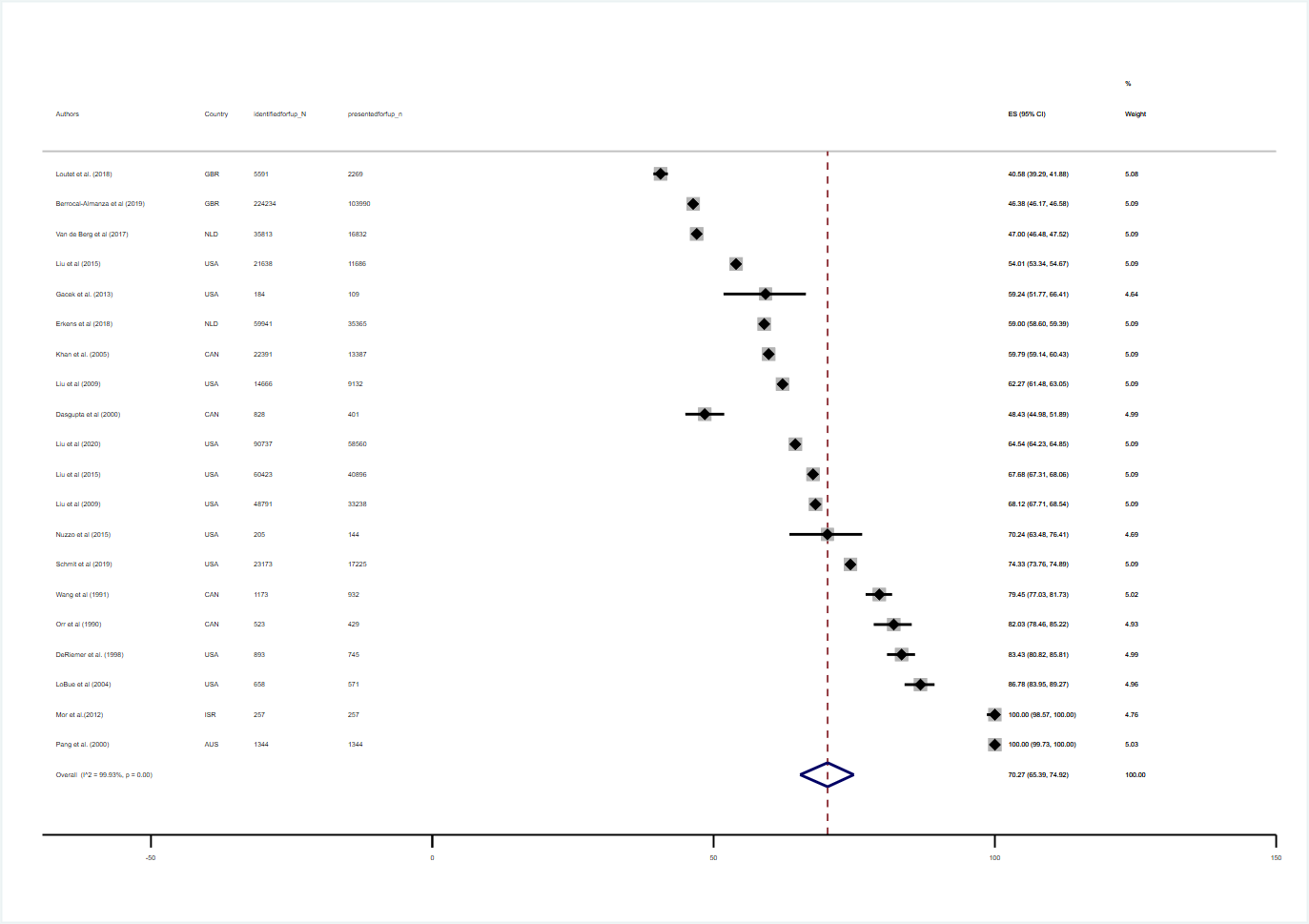
**

ES: estimate of yield, per 100.000. Diamonds: point estimates. Horizontal bands: 95 % binomial exact confidence intervals. Big hollow diamond: pooled effect.

**Fig I: Forest plot of coverage with pooled estimate**

**
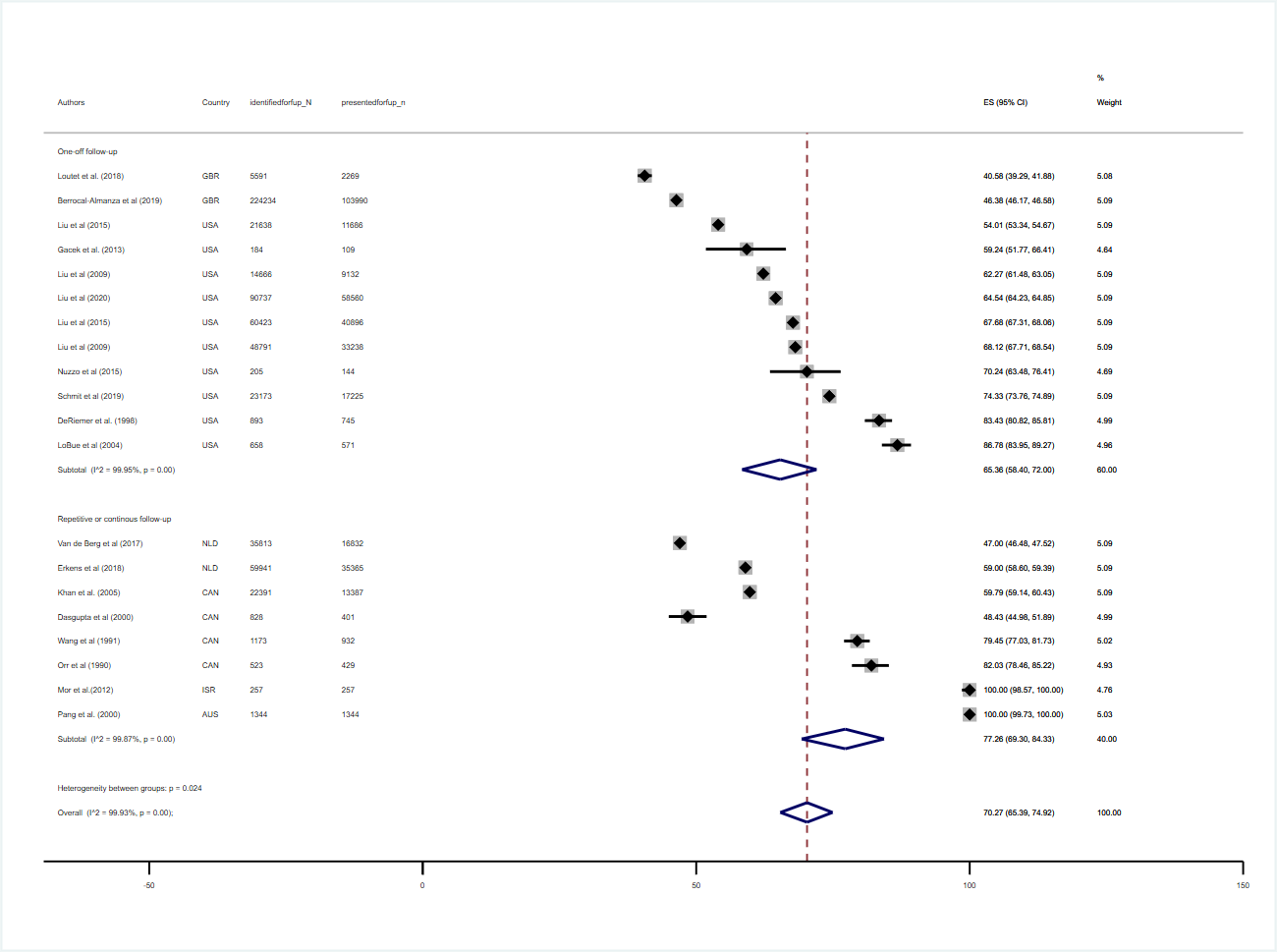
 Fig J: Forest plot of coverage with pooled estimates, stratified by programme design**

ES: estimate of yield, per 100.000. Diamonds: point estimates. Horizontal bands: 95 % binomial exact confidence intervals. Big hollow diamonds: pooled effect.

**
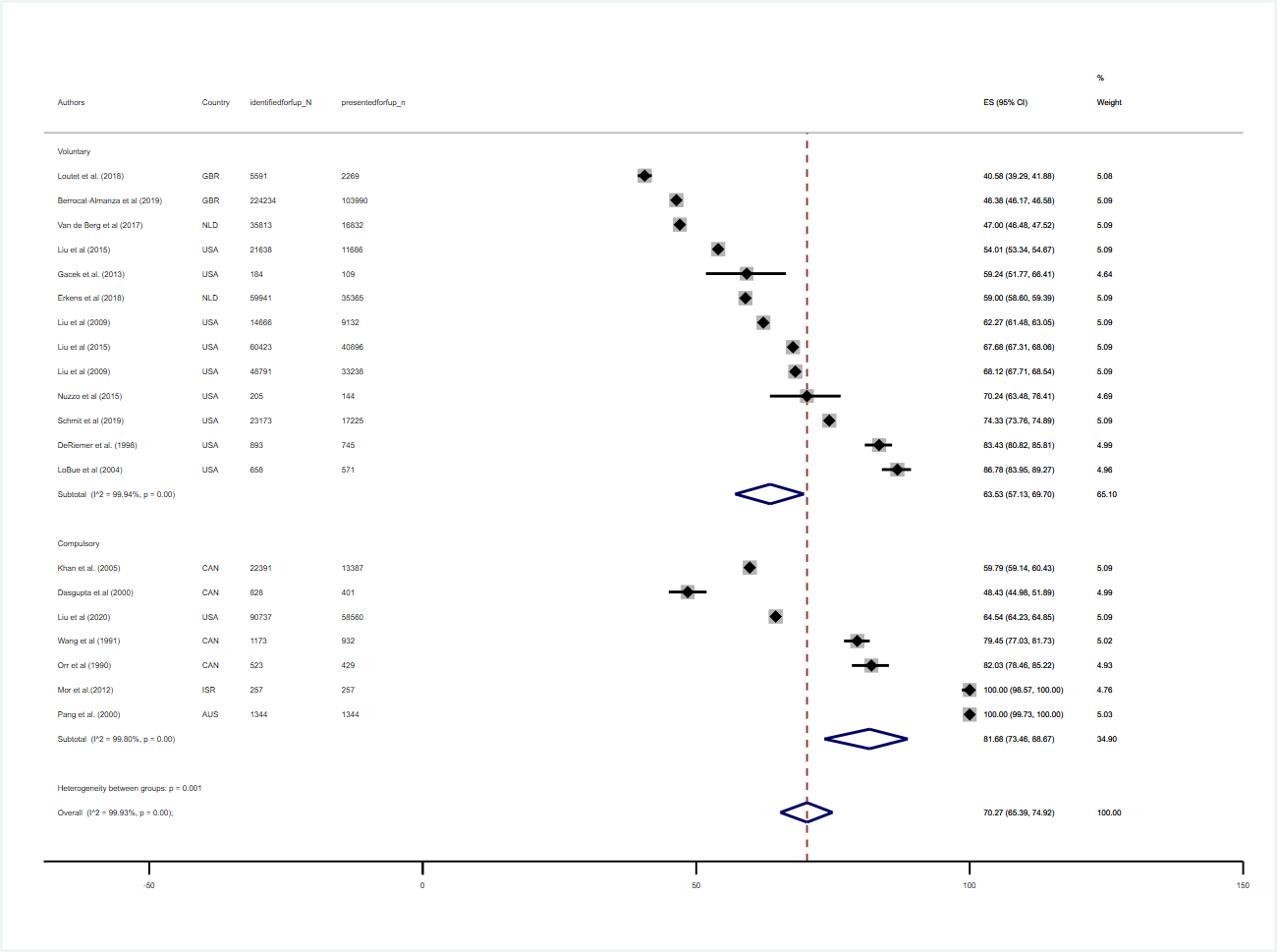
**

**Fig K: Forest plot of coverage with pooled estimates, stratified by legal schema**

ES: estimate of coverage, in %. Diamonds: point estimates. Horizontal bands: 95 % binomial exact confidence intervals. Big hollow diamonds: pooled effect.


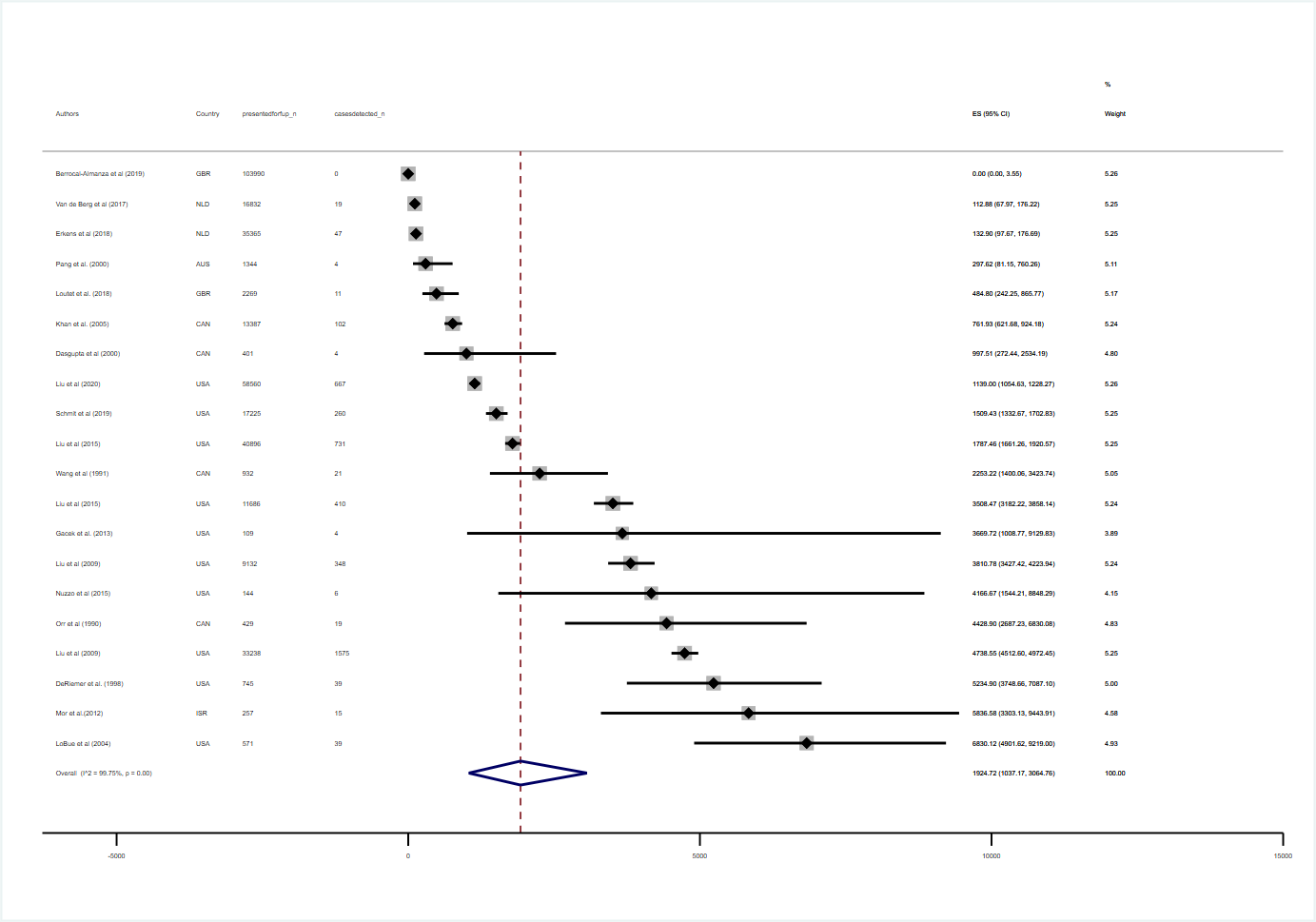


**Fig L: Forest plot of ‘screened-population-yield’ with pooled estimate**

ES: estimate of yield, per 100.000. Diamonds: point estimates. Horizontal bands: 95 % binomial exact confidence intervals. Big hollow diamonds: pooled effect.


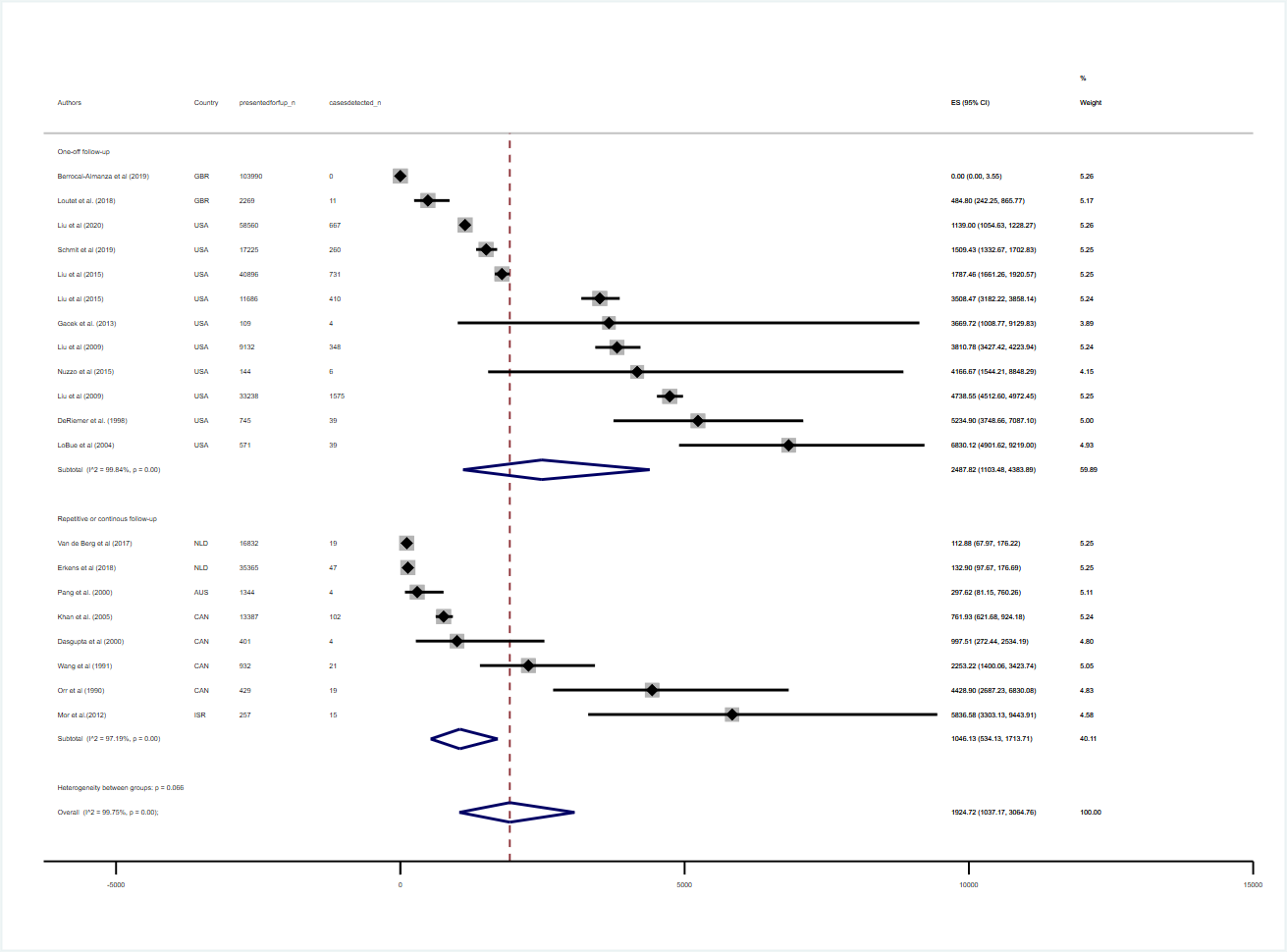


ES: estimate of yield, per 100.000. Diamonds: point estimates. Horizontal bands: 95 % binomial exact confidence intervals. Big hollow diamonds: pooled effect.

**Fig M: Forest plot of ‘screened-population-yield’ with pooled estimates, stratified by programme character**

**
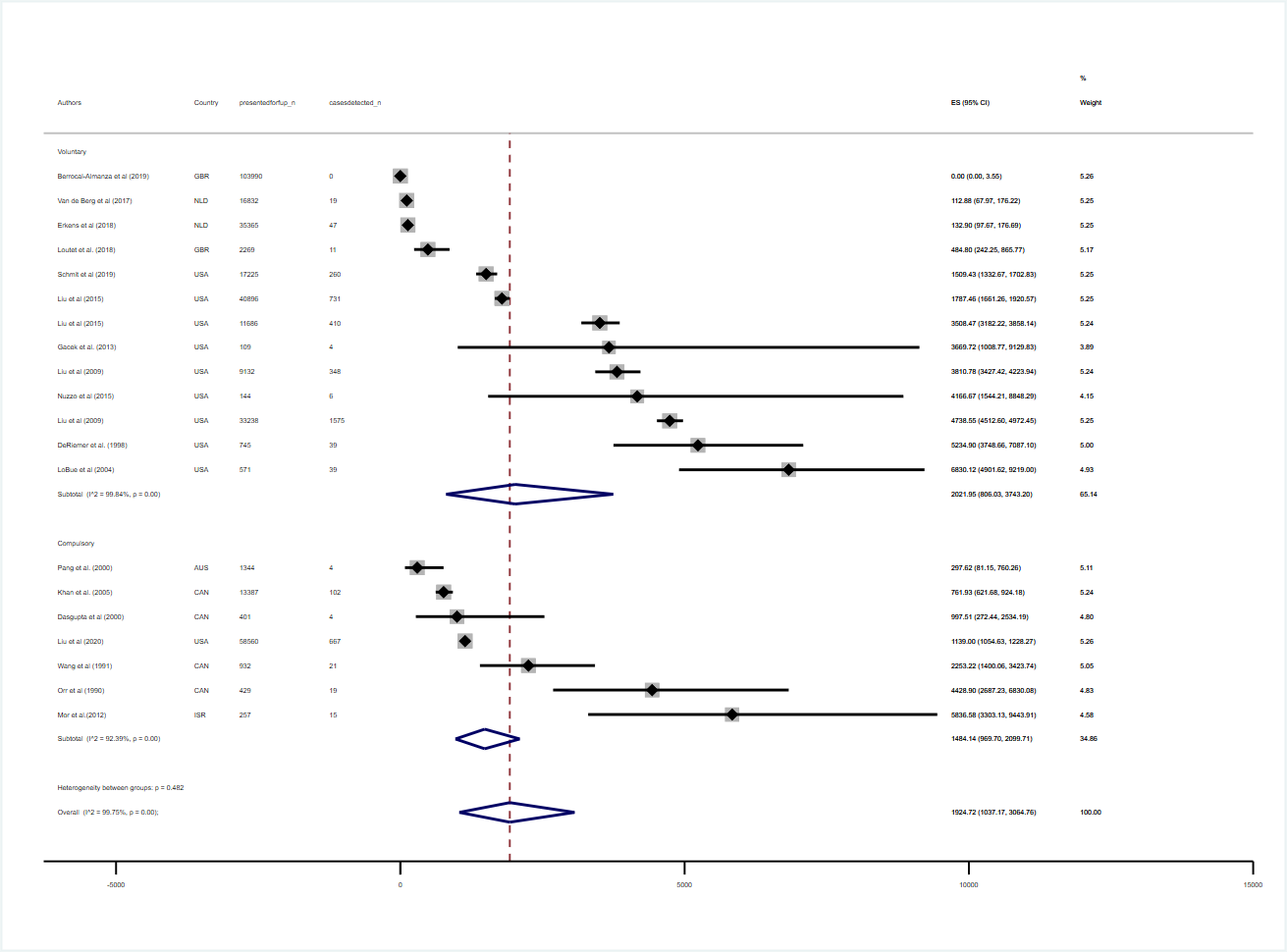
**

ES: estimate of yield, per 100.000. Diamonds: point estimates. Horizontal bands: 95 % binomial exact confidence intervals. Big hollow diamonds: pooled effect.

**Fig N: Forest plot of ‘screened-population-yield’ with pooled estimates, stratified by legal schema**

**
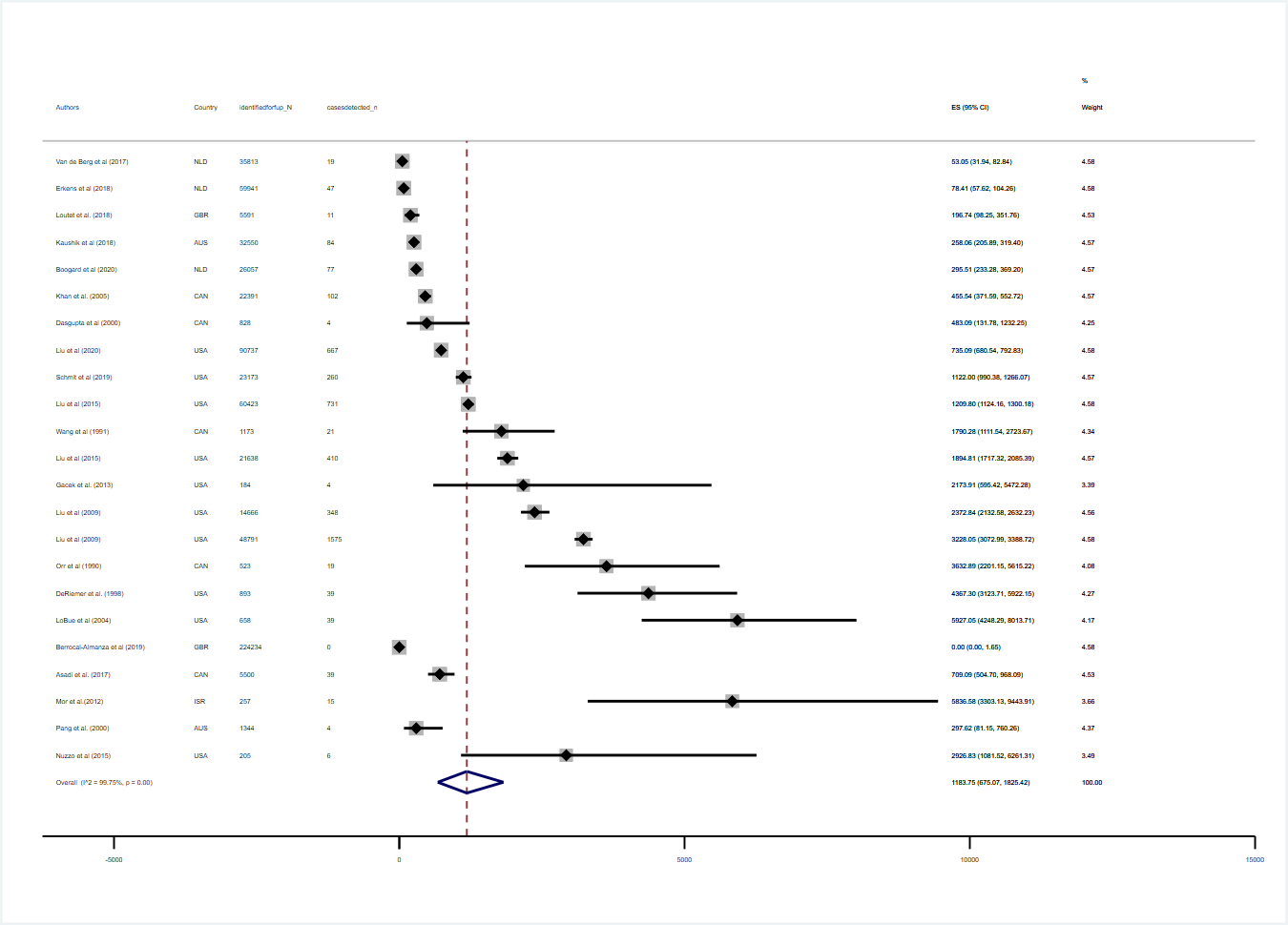
**

**Fig O: Forest plot of ‘eligible-population-yield’ with pooled estimate**

ES: estimate of yield, per 100.000. Diamonds: point estimates. Horizontal bands: 95 % binomial exact confidence intervals. Big hollow diamonds: pooled effect.


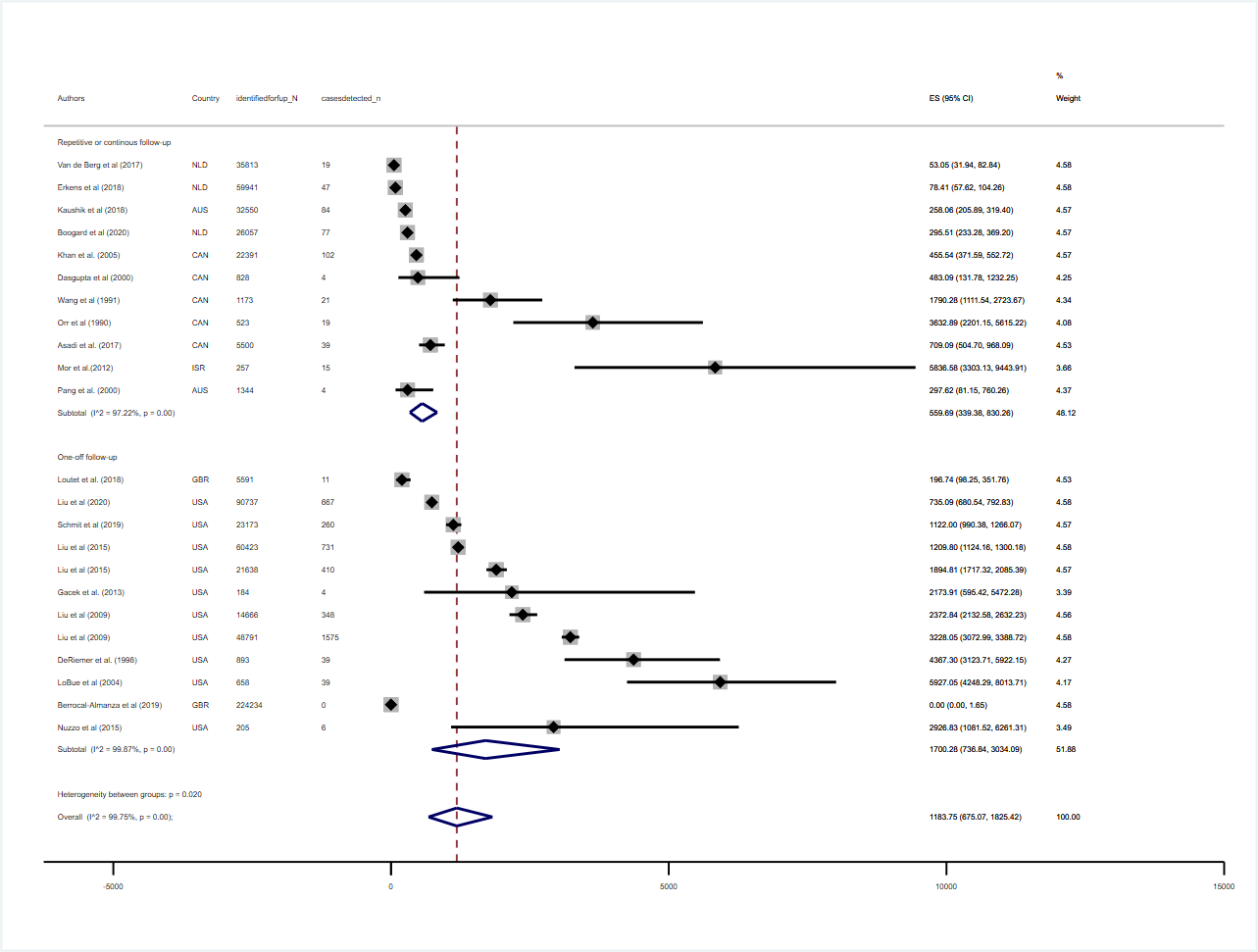


ES: estimate of yield, per 100.000. Diamonds: point estimates. Horizontal bands: 95 % binomial exact confidence intervals. Big hollow diamonds: pooled effect.

**Fig P: Forest plot of ‘eligible-population-yield’ with pooled estimates, stratified by programme character**


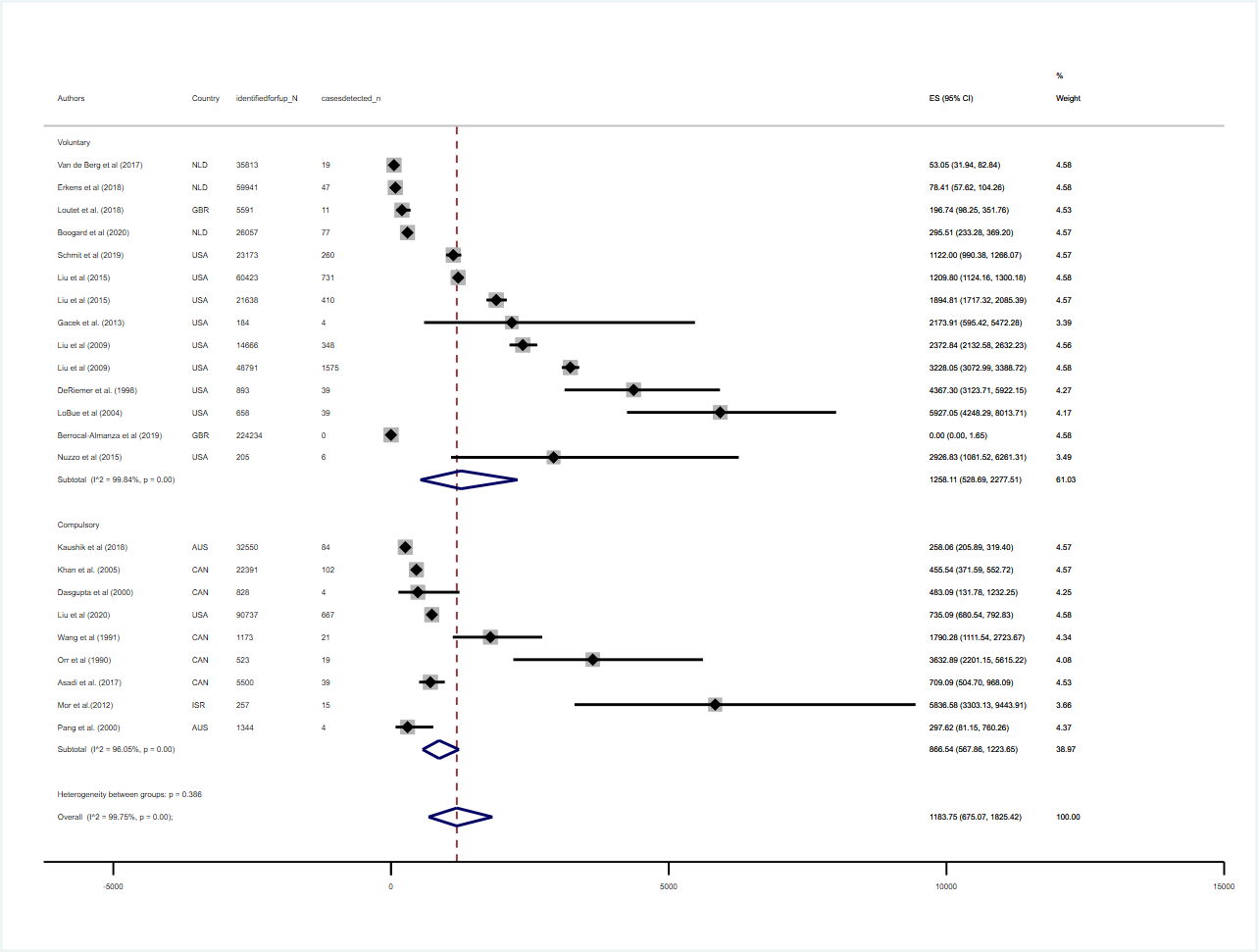


**Fig Q: Forest plot of ‘eligible-population-yield’ with pooled estimates, stratified by legal character**

ES: estimate of yield, per 100.000. Diamonds: point estimates. Horizontal bands: 95 % binomial exact confidence intervals. Big hollow diamonds: pooled effect.


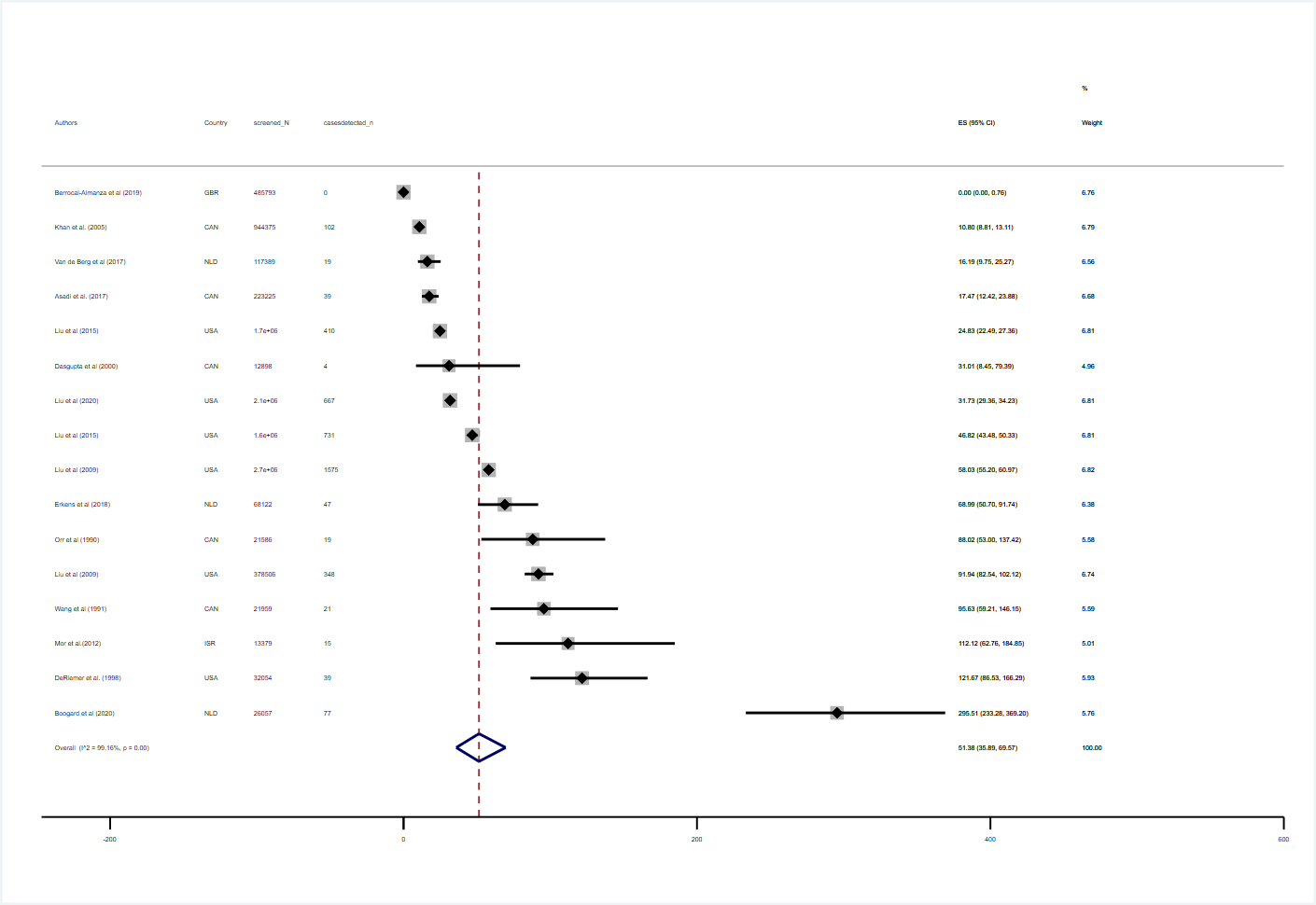


**Fig R: Forest plot of ‘whole-population-yield’ with pooled estimate**

ES: estimate of yield, per 100.000. Diamonds: point estimates. Horizontal bands: 95 % binomial exact confidence intervals. Big hollow diamond: pooled effect.


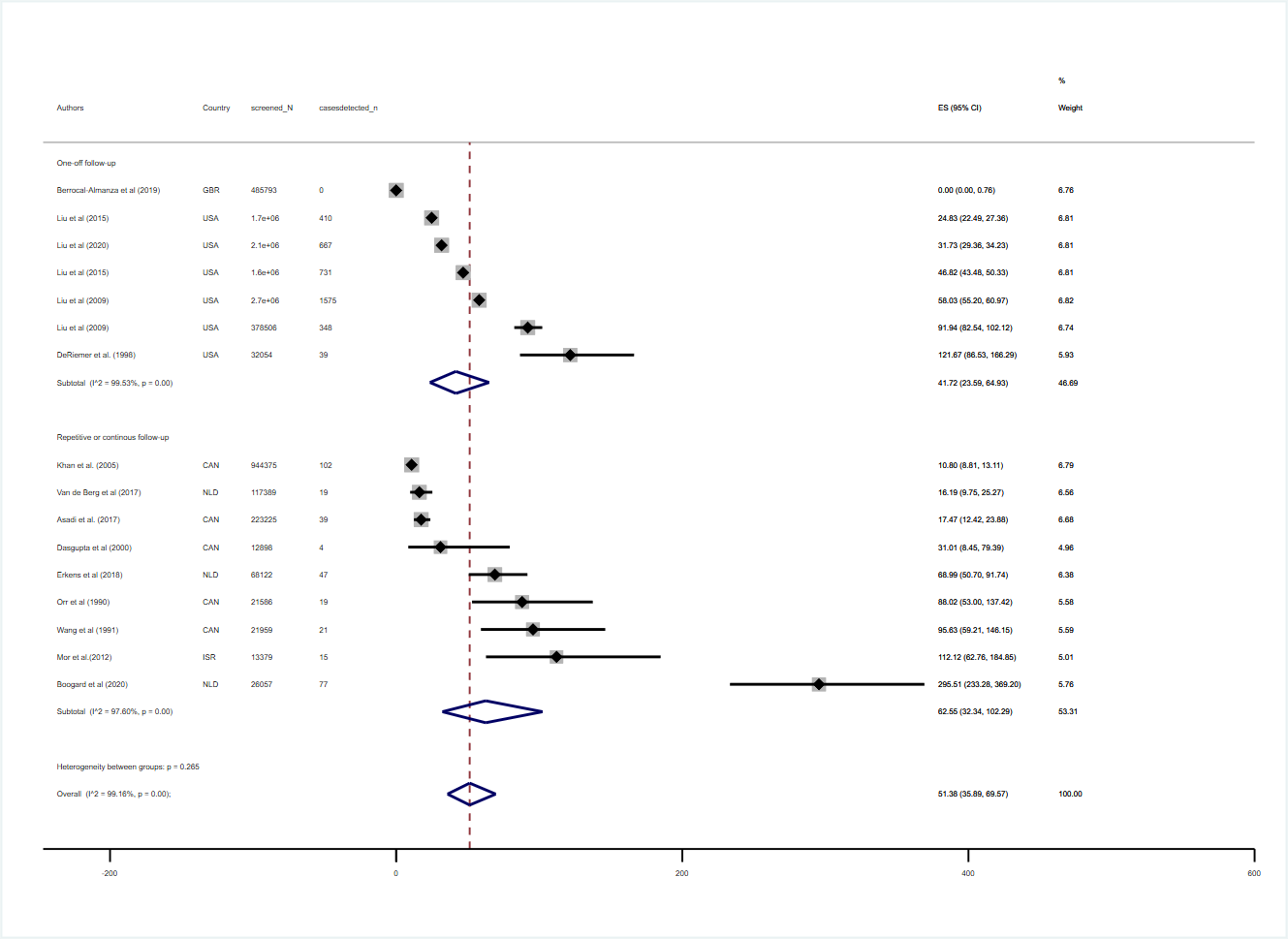


**Fig S: Forest plot of ‘whole-population-yield’ with pooled estimates, stratified by programme character**

ES: estimate of yield, per 100.000. Diamonds: point estimates. Horizontal bands: 95 % binomial exact confidence intervals. Big hollow diamonds: pooled effect.


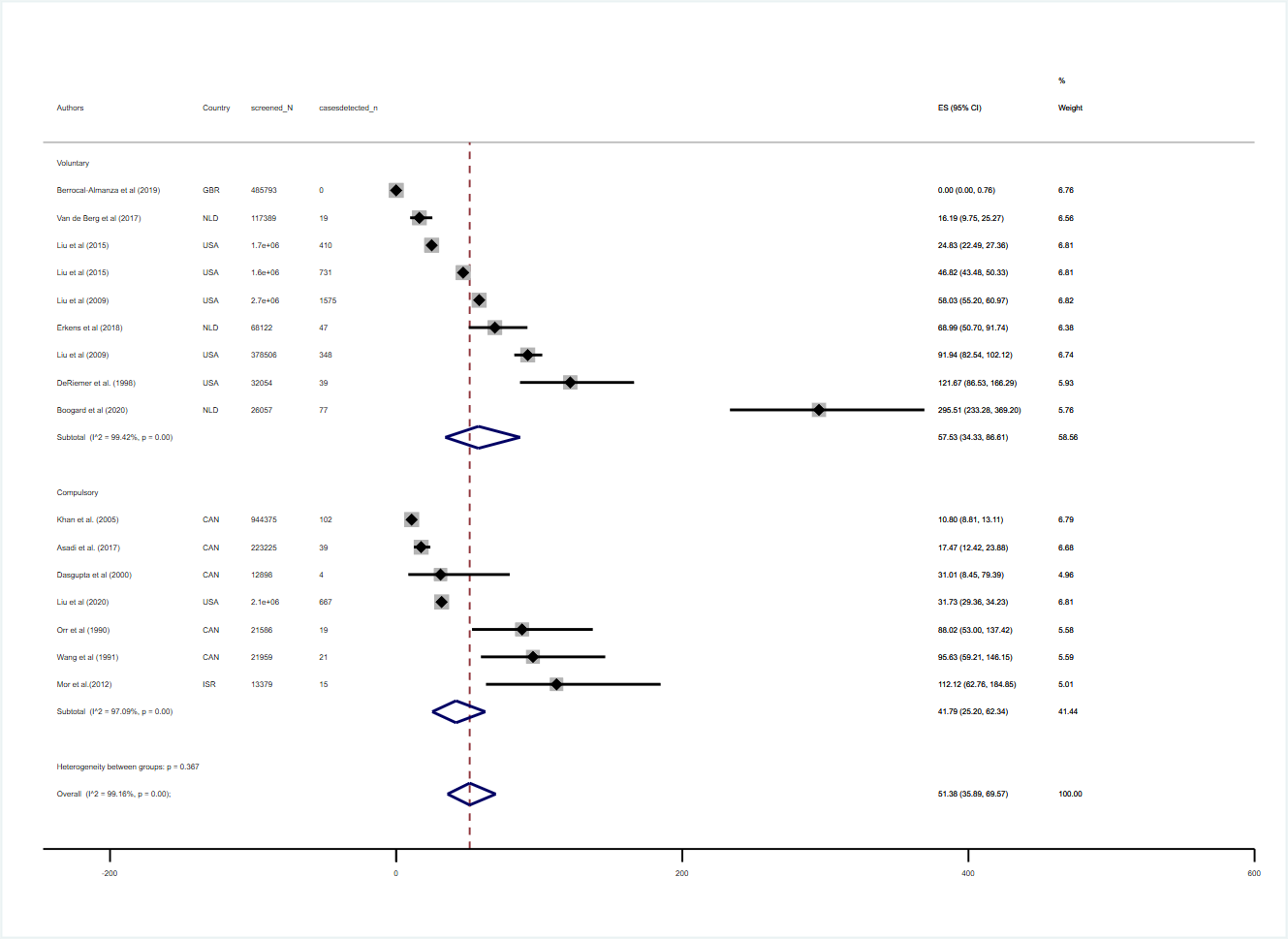


**Fig T: Forest plot of ‘whole-population-yield’ with pooled estimates, stratified by legal schema**

ES: estimate of yield, per 100.000. Diamonds: point estimates. Horizontal bands: 95 % binomial exact confidence intervals. Big hollow diamonds: pooled effect.

5 References

1. Van der Werf MJ, Lönnroth K. Pre-entry, post-entry, or no tuberculosis screening? The Lancet Infectious Diseases. 2014;14: 1171–1172. doi:10.1016/S1473-3099(14)70998-3

2. Chan IHY, Kaushik N, Dobler CC. Post-migration follow-up of migrants identified to be at increased risk of developing tuberculosis at pre-migration screening: a systematic review and meta-analysis. The Lancet Infectious Diseases. 2017;17: 770–779. doi:10.1016/S1473-3099(17)30194-9

3. Harstad I, Heldal E, Steinshamn SL, Garåsen H, Jacobsen GW. Tuberculosis screening and follow-up of asylum seekers in Norway: a cohort study. BMC Public Health. 2009;9. doi:10.1186/1471-2458-9-141

4. Nyaga VN, Arbyn M, Aerts M. Metaprop: a Stata command to perform meta-analysis of binomial data. Arch Public Health. 2014;72: 39. doi:10.1186/2049-3258-72-39

5. Deeks JJ, Higgins, Julian PT, Altman DG. Chapter 9: Analysing data and undertaking meta-analyses. Version 5.1.0 [updated March 2011]. Cochrane Handbook for Systematic Reviews of Interventions. Version 5.1.0 [updated March 2011]. The Cochrane Collaboration 2011; Available: https://handbook-51.cochrane.org/chapter_9/9_5_2_identifying_and_measuring_heterogeneity.htm
